# Patterning the Asteraceae Capitulum: Duplications and Differential Expression of the Flower Symmetry *CYC2*-Like Genes

**DOI:** 10.3389/fpls.2018.00551

**Published:** 2018-04-25

**Authors:** Jie Chen, Chu-Ze Shen, Yan-Ping Guo, Guang-Yuan Rao

**Affiliations:** ^1^School of Life Sciences, Peking University, Beijing, China; ^2^Ministry of Education Key Laboratory for Biodiversity Science and Ecological Engineering and College of Life Sciences, Beijing Normal University, Beijing, China

**Keywords:** Asteraceae, capitulum, *CYC2*-like genes, divergence in function, gene duplication, ray floret

## Abstract

There are several types of capitulum in the Asteraceae due to different combinations of florets varying in corolla shape and stamen development. Previous studies have shown that the formation of ray florets on a radiate capitulum may be related to the parallel co-option of *CYC2*-like genes among independent Asteraceae lineages. The present work tests that hypothesis and attempts to shed light on the pattern of evolution of the Asteraceae capitulum and floral heteromorphism under the regulation of *CYC2*-like genes. In this study, the evolutionary history of *CYC2*-like genes in the Asterales was reconstructed and their expression patterns were examined in species representing different capitulum types and several major Asteraceae lineages. To clarify the role of CYC2d clade genes in morphogenesis of ray flowers, overexpression of *ClCYC2d* was conducted in *Chrysanthemum lavandulifolium*. Our results show that there are six *CYC2*-like members in the Asteraceae; they are results of five duplication events starting from a single-copy gene in the common ancestor of the Goodeniaceae-Calyceraceae-Asteraceae group and completing before the divergence of the subfamily Carduoideae of Asteraceae. Spatial expression pattern of each of the Asteraceae *CYC2*-like members is conserved across the family. All the six members contribute to the development of the complexity of a capitulum: To form a ray floret, either *CYC2c* or *CYC2g* plays an essential role, while *CYC2d* represses the development of dorsal corolla lobes and stamens of the floret. In sum, the developmental program of making a ray flower is conserved involving functionally divergent *CYC2*-like genes. Based on extensive species sampling, this study provides an overview of the mode of regulation of *CYC2*-like genes that patterns the capitulum architectures and their transitions.

## Introduction

Morphological and structural innovations of flowers and inflorescences are intimately linked to the reproduction and adaptation of angiosperms (Endress, 2011; Kirchoff and Claßen-Bockhoff, 2013; Teo et al., 2014). In particular, changes in floral organ number and morphology as well as varied arrangements of flowers in an inflorescence influence the plant-pollinator interaction and reproductive success (Wyatt, 1982; Kalisz et al., 2006; Ushimaru et al., 2009; Harder and Prusinkiewicz, 2013). Several angiosperm families, mainly the recently diverged lineages, exhibit a tendency to maximize reproductive efficiency by condensing heterogeneous flowers into a head-like inflorescence (Harris, 1999; Reuther and Claßen-Bockhoff, 2013). The evolution of such head-like inflorescences is usually correlated with the morphology and the arrangement of their component flowers (Classen-Bockhoff, 1990; Coen and Nugent, 1994).

The inflorescence of Asteraceae, one of the largest and most successful families of flowering plants, is a specialized, complex structure called the capitulum. This structure consists of, in most cases, many florets that are highly compressed and that resemble a solitary flower (Gillies et al., 2002; Jeffrey, 2009; Zhao et al., 2016). The florets are variable in morphology mainly in terms of their corolla symmetry and fertility such that they are classified into several types: actinomorphic tubular florets (usually bisexual), zygomorphic ray florets (usually female/neutral), zygomorphic ligulate florets (bisexual), zygomorphic bilabiate florets and asymmetric pseudobilabiate florets (female/neutral). Different combinations of different florets give rise to a variety of capitulum types (Jeffrey, 1977, 2009; Bremer, 1994). In many cases, a heterogeneous capitulum has a flat radiate form, and its flowers differentiate into the marginal ray and the central tubular disc florets. Such differentiation makes the capitulum functionally like a single flower and is therefore termed a pseudanthium. The marginal female or neutral ray florets have been proven to enhance the ability to attract animal pollinators, thus increasing outcrossing and fitness (Leppik, 1977; Stuessy et al., 1986; Andersson and Widén, 1993; Andersson, 1999; Nielsen et al., 2002; Chapman and Abbott, 2010). In contrast to the radiate capitulum, discoid and ligulate flower heads are composed of only bisexual actinomorphic tubular and zygomorphic ligulate florets, respectively. The disciform capitulum is a relatively rare type of capitulum that is composed of disc florets in the center and a few female pseudobilabiate florets at the margin (Leppik, 1977; Harris, 1995; Jeffrey, 2009; Ren and Guo, 2015). Various types of capitulum are scattered throughout the phylogeny of the family, implying that parallel or convergent evolution may be involved in patterning the capitulum architecture (Harris, 1995; Panero and Funk, 2002, 2008; Funk et al., 2009).

Classical studies on *Senecio* and *Layia* suggested a single- or two-factor genetic model of ray floret development (Trow, 1912; Hull, 1974; Ford and Gottlieb, 1990). In *Senecio*, the single-factor model works with two linked CYCLOIDEA2-like (CYC2-like) TCP transcription factors, RAY1 and RAY2 (Kim et al., 2008). The CYC2 regulatory framework that controls the formation of floral zygomorphy has been extensively investigated in angiosperms (Luo et al., 1996, 1999; Hileman, 2014). A series of functional investigations have revealed that *CYC2*-like genes are responsible for the development of zygomorphic ray florets in Asteraceae.

In the radiate capitula of *Helianthus annuus, Gerbera hybrida, Senecio vulgaris* and *Chrysanthemum morifolium, CYC2*-like genes regulate the morphogenesis of florets and differentiation of the marginal and central layer(s) of florets (Broholm et al., 2008; Kim et al., 2008; Chapman et al., 2012; Juntheikki-Palovaara et al., 2014; Garcês et al., 2016; Huang et al., 2016). The differentiated expression patterns and selection regimes of *CYC2*-like paralogs showed close correlations with functional divergence (Chapman et al., 2008; Tähtiharju et al., 2012). Changes in the expression of some *CYC2*-like members led to gradient transitions between ray and disc florets, including modifications of ray-specific characteristics, such as the zygomorphy of the corolla, length of the ligule and development of the stamen (Fambrini et al., 2003; Broholm et al., 2008; Chapman et al., 2012; Juntheikki-Palovaara et al., 2014; Huang et al., 2016); some other *CYC2*-like members influence only the morphs of the marginal ray florets (Kim et al., 2008; Garcês et al., 2016).

Chapman et al. (2012) analyzed the evolution of *CYC2*-like genes using several Asteraceae species in which radiate capitula arose independently. Their study indicated that different *CYC2*-like paralogs might have been co-opted and lost independently in certain lineages of the Asteraceae. Thus far, however, the history of duplication and the relationships of the CYC2 clade genes in the Asteraceae remain unresolved due to sampling problems. On the one hand, previous studies have focused on radiate species only or involved a few distantly related species samples in the family; on the other hand, their phylogenetic analyses were often based on a region between the TCP and R domains of *CYC2*-like genes (Kim et al., 2008; Chapman et al., 2012; Tähtiharju et al., 2012; Huang et al., 2016; Bello et al., 2017). Moreover, the evidence that co-option of different *CYC2*-like copies occurred independently in less-related species per se is inadequate for determining the reason why various Asteraceae capitula exhibit a complex pattern of evolution.

Hence, in the present study, we explore Asteraceae capitulum evolution by phylogenetic, expression and functional analyses of the CYC2 clade genes. Our phylogenetic analysis was based on sampling a large number of species covering all major lineages and all four major capitulum types of the family and sequencing the full-length of the genes. We aim to first, untangle the evolutionary trajectories of *CYC2*-like genes in the Asteraceae; second, shed light on the correlation between the functional diversification of the *CYC2*-like genes and the evolution of ray florets and capitulum architecture; and finally, explore the roles of the gain and loss of *CYC2*-like genes in the transformation of diverse florets and capitula within and among lineages during Asteraceae evolution.

## Materials and methods

### Plant sampling

**Figure 1:**
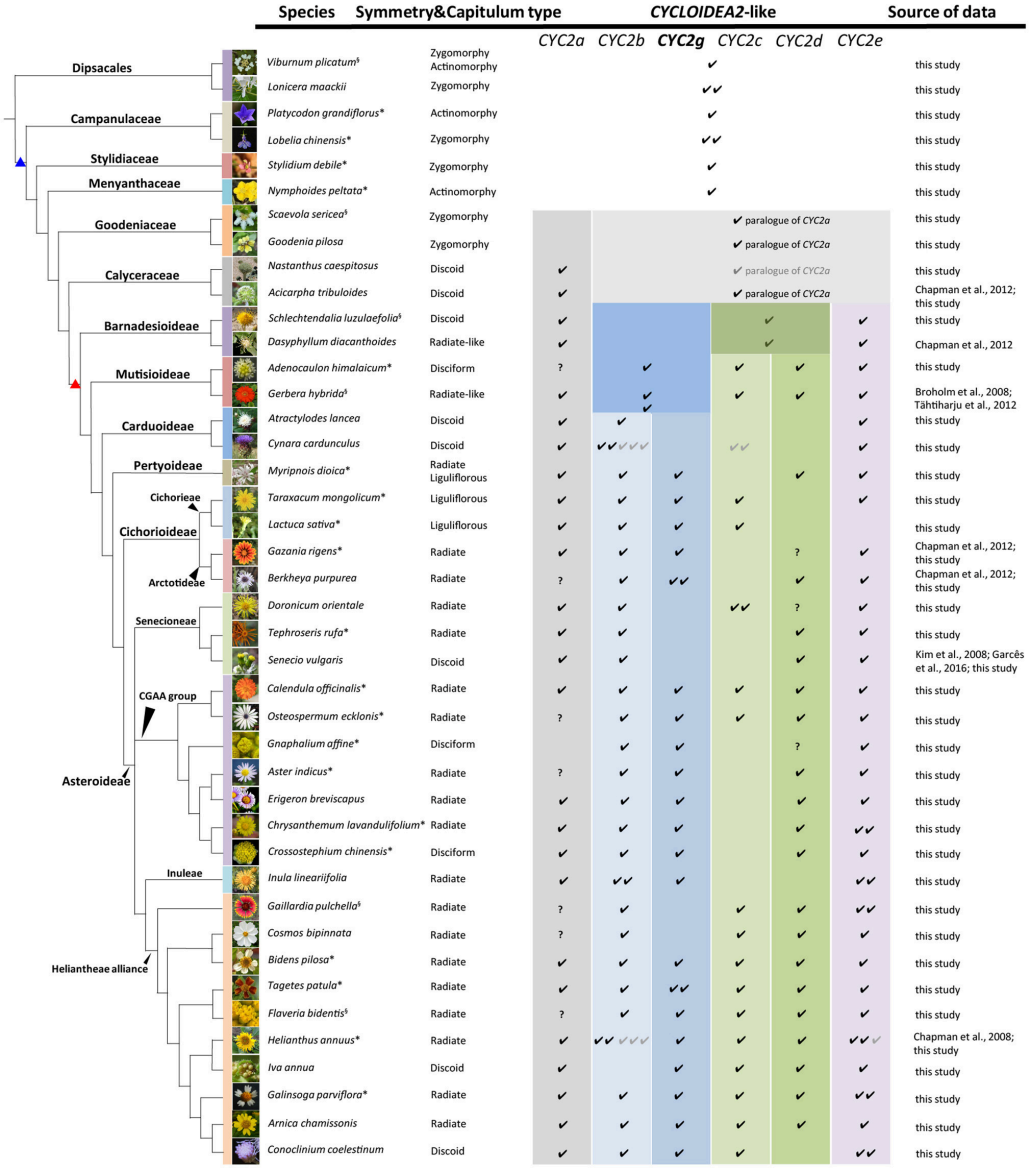
Capitulum morphologies of the representative sampled species in this and previous studies. The species are listed according to the phylogenetic relationships reported by Panero and Funk (2008), Funk et al. (2009) and the APG IV (Chase et al., 2016). The branches marked with blue and red triangles stand for the Asterales and Asteraceae, respectively. The presence of each *CYC2*-like gene in each species is marked by “√,” the gray “√” indicates a pseudogene, and “?” means the gene has not been isolated so far in this species but is proved to exist in other relatives of the same tribe. The *CYC2g* clade gene highlighted in bold letters is newly identified and named in this study. The superscript on the top right of each species name denotes the source of the flora image: ^*^ stands for photographs taken by the present authors, “§” stands for photos from Wikipedia (https://www.wikipedia.org/), and the rest are from several open websites: *Lonicera maackii* (http://www.eggert-baumschulen.de), *Goodenia pilosa* (http://www.northqueenslandplants.com), *Nastanthus caespitosus* (http://www.birdingpatagonia.com), *Acicarpha tribuloides* (http://www.nippon-shinyaku.co.jp), *Dasyphyllum diacanthoides* (http://www.chlorischile.cl), *Atractylodes lancea* (http://flowers.la.coocan.jp), *Cynara cardunculus* (https://upload.wikimedia.org), *Berkheya purpurea* (http://www.jardinexotiqueroscoff.com), *Doronicum orientale* (http://luirig.altervista.org), *Senecio vulgaris* (http://ichn.iec.cat), *Erigeron breviscapus* (http://www.cgi.gov.cn), *Inula lineariifolia* (http://flora.nhm-wien.ac.at), *Cosmos bipinnata* (http://www7a.biglobe.ne.jp/~flower_world/images/Cosmos%20bipinnatus/DSC03863.JPG), *Iva annua* (http://www.alabamaplants.com), *Arnica chamissonis* (http://www1.lf1.cuni.cz), *Conoclinium coelestinum* (http://www.asergeev.com).

Fifty-nine species across the major lineages of Asterales were sampled for phylogenetic analysis of the Asteraceae CYC2 clade genes. Of the 59 species samples, 50 samples were collected from 20 tribes of seven subfamilies within the Asteraceae (Panero and Funk, 2008; Funk et al., 2009; Mandel et al., 2015), and nine were collected from the allied families, that is, Calyceraceae, Goodeniaceae, Menyanthaceae, Stylidiaceae, and Campanulaceae (Chase et al., 2016). In addition, two species from Adoxaceae and Caprifoliaceae (Dipsacales) were sampled and used as the outgroup (Figure 1). Most of the samples were collected from natural populations or cultivated plants, while others were germinated from seeds bought from international seed banks. Living plants were grown in the greenhouse of Peking University (Beijing, China) until flowering. The detailed sampling information is listed in Table S1.

### DNA/RNA extraction and isolation of the CYC2 clade genes

Genomic DNA was extracted from silica-gel-dried or fresh leaves with a Plant Genome Extraction Kit following the manufacturer's protocol (Tiangen Biotech, Beijing, China). Total RNA was extracted by TRIzol reagent (Invitrogen, USA) from inflorescences or florets prepared in liquid nitrogen. The first-strand cDNA was synthesized using SuperScript^TM^ III Reverse Transcriptase (Invitrogen, USA) by a tagged oligo(dT)_18_ primer (Table S2). To isolate the *CYC2*-like genes, 3′-RACE PCR, 5′-RACE PCR and genome-walking methods were carried out. First, *CYC2*-like sequences of *G. hybrida, H. annuus* and *S. vulgaris* downloaded from the NCBI database (Table S1) were aligned to design primers. As a result, four conserved Asteraceae primers were developed. Second, the two forward primers, CYC2-3rF1 and CYC2-3rF2, positioned in the TCP domain, and two universal reverse primers, GSP1 and GSP2, were used for 3′-RACE PCR in most of the studied species. All PCR products were ligated into pGEM-T vectors (Promega, Madison, USA) and cloned, and then at least 24–48 positive clones were sequenced. Third, a species-specific reverse primer for each copy was designed according to the obtained 3′-end sequences and combined with the conserved forward primer CYC2-ATG, starting from the start codon to amplify the full-length of the *CYC2* sequences (In the case of *CYC2a* genes, a specific forward primer, CYC2a-ATG, was used instead of CYC2-ATG). When CYC2 (a)-ATG primers did not work, 5′-RACE PCR (Invitrogen, USA) was used to acquire the rest of the CDS. In addition, some species had only dried leaf materials, or their *CYC2*-like members may not be expressed; in this case, a partial sequence was first obtained using the degenerated primer CYC2-ATG or CTf paired with CTr, as reported by Chapman et al. (2008), and then, genome walking was modified from Balavoine (1996) or performed with a GenomeWalker Universal kit (BD Biosciences, USA) was conducted to obtain a full-length gene sequence. All the conserved or universal primers are listed in Table S2.

For some species of interest, segments or short reads of the *CYC2*-like genes were retrieved by BLASTn and assembled together from the public next-generation sequencing (NGS) data in GenBank (accession numbers recorded in Table S1) under the condition that these contigs have high similarities to the queries from close relatives used in this study to avoid disruption caused by false alignment.

### Phylogenetic reconstruction

In total, 192 *CYC2*-like sequences were obtained and used in the phylogenetic analysis. Most of the sequences are of the complete CDS, and a few cover > 90% of the region of the CDS; however, two exceptions, *VplCYC2* and *PgrCYC2*, only contain a partial sequence from N-terminal to the R domain. Protein alignment was conducted by the online toolkit webPRANK (https://www.ebi.ac.uk/goldman-srv/webprank/, Löytynoja and Goldman, 2010) and was adjusted by hand in BioEdit (Hall, 1999). The aligned sequences are provided as Supplementary Material.

The best-fit evolutionary model was derived from ProtTest 3.4 (updated in 2014; Darriba et al., 2011). Maximum likelihood (ML) analysis was run in RAxML v8.2.9 (Stamatakis, 2014) with 1000 bootstrap replicates. Bayesian inference was performed in MrBayes v.3.2.1 (Ronquist and Huelsenbeck, 2003) with 2,000,000 generations, sampling every 1,000 generations and taking the first 25% of the trees as burn-in. Sequences of the Adoxaceae and Caprifoliaceae species were used as the outgroup to root the Asterales *CYC2*-like gene tree. To reconstruct a robust and well-resolved phylogeny of the Asteraceae *CYC2*-like genes, we conducted the phylogenetic analyses using four sub-datasets containing different regions of the protein sequences.

### Gene structure, motif identification and genomic localization of the asteraceae CYC2 clade genes

Gene structures were determined by comparing the transcript sequences with the genomic sequences. To elucidate characteristics of the conserved motifs of the Asterales CYC2 proteins, the web-based tool Multiple Expectation Maximization for Motif Elicitation (MEME) v.4.11.2 was employed (http://meme-suite.org/tools/meme, Bailey et al., 2009).

Because four released genome datasets, *Cynara cardunculus* var. *scolymus* (Scaglione et al., 2016), *Lactuca sativa* (Reyes-Chin-Wo et al., 2017), *H. annuus* (Badouin et al., 2017), and *Erigeron canadensis* (*Conyza canadensis*) (Peng et al., 2014), are available for linkage background analysis, the duplication events of *CYC2*-like genes occurred in the Asteraceae can be clarified. The websites are listed in Table S1. From the genome data, *CYC2* homologs and their localities were retrieved and are documented in Table S3. We inferred the phylogeny of these contigs of *CYC2*-like genes by comparing them with the homologs of several other species.

### Expression of *CYC2*-like genes among distant species in the asteraceae

The expression patterns of all *CYC2*-like genes in the marginal ray and the adjacent disc florets were comparatively analyzed to uncover roles of different *CYC2*-like genes in determining characters of the zygomorphic ray versus the actinomorphic disc floret among different Asteraceae lineages. Total RNA was extracted as aforementioned and then normalized to a concentration of 50–100 ng μl^−1^. Then, cDNA was synthesized by a two-step RT-PCR system (Tiangen, China) with random primers and diluted twice with double-distilled water. RT-qPCR was carried out by the 7500/7500 Fast Real-Time PCR System (Applied Biosystems) following the two-step program. The reaction system was in a volume of 20 μl and contained 1 μl cDNA template using the SYBR *Premix Ex Taq*^TM^ (Tli RNaseH Plus) system (TaKaRa, Japan). The primer information is provided in Table S4. Transcript levels were normalized against that of *ACTIN7*. Three biological and technical replicates per sample were applied to avoid random errors. The results are presented as the means ± standard error (s.e.) of three biological repeats. Statistical analysis and graphs were performed in GraphPad Prism 5 (GraphPad software) using two-sample tailed Student's *t*-test after testing the homogeneity of variance.

### Plant transformation and detection of transgenic lines

Considering the phenotypic inconsistency of *CYC2d* overexpression in *G. hybrida* and *S*. *vulgaris* (Kim et al., 2008; Juntheikki-Palovaara et al., 2014), we conducted a similar transformation experiment in *C*. *lavandulifolium*. The overlapping PCR strategy was used to bridge the CaMV 35s promoter, *ClCYC2d* coding region and 35s polyA terminator to construct the binary vector. The transformation system was modified from the protocol used for *Chrysanthemum indicum* (Ledger et al., 1991). The overlapped fragment with *Kpn*I and *BamH*I enzyme sites was cloned into a modified pCAMBIA2300 backbone and then transformed into *C. lavandulifolium* through the *Agrobacterium* strain GV3101 (Spena et al., 1987; Mitiouchkina and Dolgov, 2000).

As explants, sterilized young leaf pieces were cut into pieces. After one day of preculture on 1/2 MS medium [for 1 liter: 2.2 g MS salt (Sangon, China), 250 mg timentin (Sangon, China), pH 5.7], these explants were soaked in a 5% sucrose solution of *Agrobacterium* with OD_600_ = 0.8–1.0 containing 10 μM acetosyringone for 20–25 min. Then, those explants were dried with sterile absorbent paper, cultured on 1/2 MS medium in darkness for 2 days, were washed in sterilized water and placed on selection MS medium (containing 0.2 mg l^−1^ 6-BA, 0.1 mg l^−1^ NAA, 20–30 mg l^−1^ kanamycin, and 250 mg l^−1^ timentin, pH 5.7). The medium was replaced by fresh medium every 2 weeks until there were small plantlets or calli; the concentration of kanamycin (10 mg l^−1^) was increased every time the medium was refreshed until it reached 30 mg l^−1^. The plantlets were planted in rooting medium (1/2 MS medium containing 0.05 mg l^−1^ NAA, 30 mg l^−1^ kanamycin, and 250 mg l^−1^ carbenicillin, pH 5.7) for one month and were then transferred to soil. The whole process of tissue culture was under a 16 h light/8 h dark cycle and a temperature of 25°C. Leaves of the rooted plantlets were used to detect positive individuals using the PLACF forward primer paired with the LACR reverse primer in genomic DNA and the capitulum issues were detected by a ClCYC2d-specific forward primer (ClCYC2d-sf) paired with a 35s polyA reverse primer (35spA-r) in transcripts (Table S5).

Positive individuals were detected for the expression of *CYC2*-like genes and capitulum morphology in comparison with the wildtype individuals. The primers used during this experiment are listed in Table S4. The lengths of ray flowers and diameters of capitula were calculated on 10 flower heads per line.

## Results

### Sequence characters of *CYC2*-like genes and their phylogenetic relationships

During this study, 158 *CYC2*-like genes were isolated with their sequences deposited in the GenBank (Accession numbers MG593365–MG593522). In addition, 34 contigs were assembled from the genome and transcriptome data in the GenBank; 28 *CYC2*-like homologs were retrieved from the genome sequencing data of *Cy. cardunculus* var. *scolymus* (Scaglione et al., 2016), *L. sativa* (Reyes-Chin-Wo et al., 2017), *H. annuus* (Badouin et al., 2017), and *E. canadensis* (Peng et al., 2014) (see Table S1 for their accession IDs and Figure S1 for their mapping); together with the previously reported genes, in total 243 *CYC*2-like sequences were identified from 59 species of Asterales and two outgroup species of Dipsacales (Figure 1 and Table S1).

**Figure 2:**
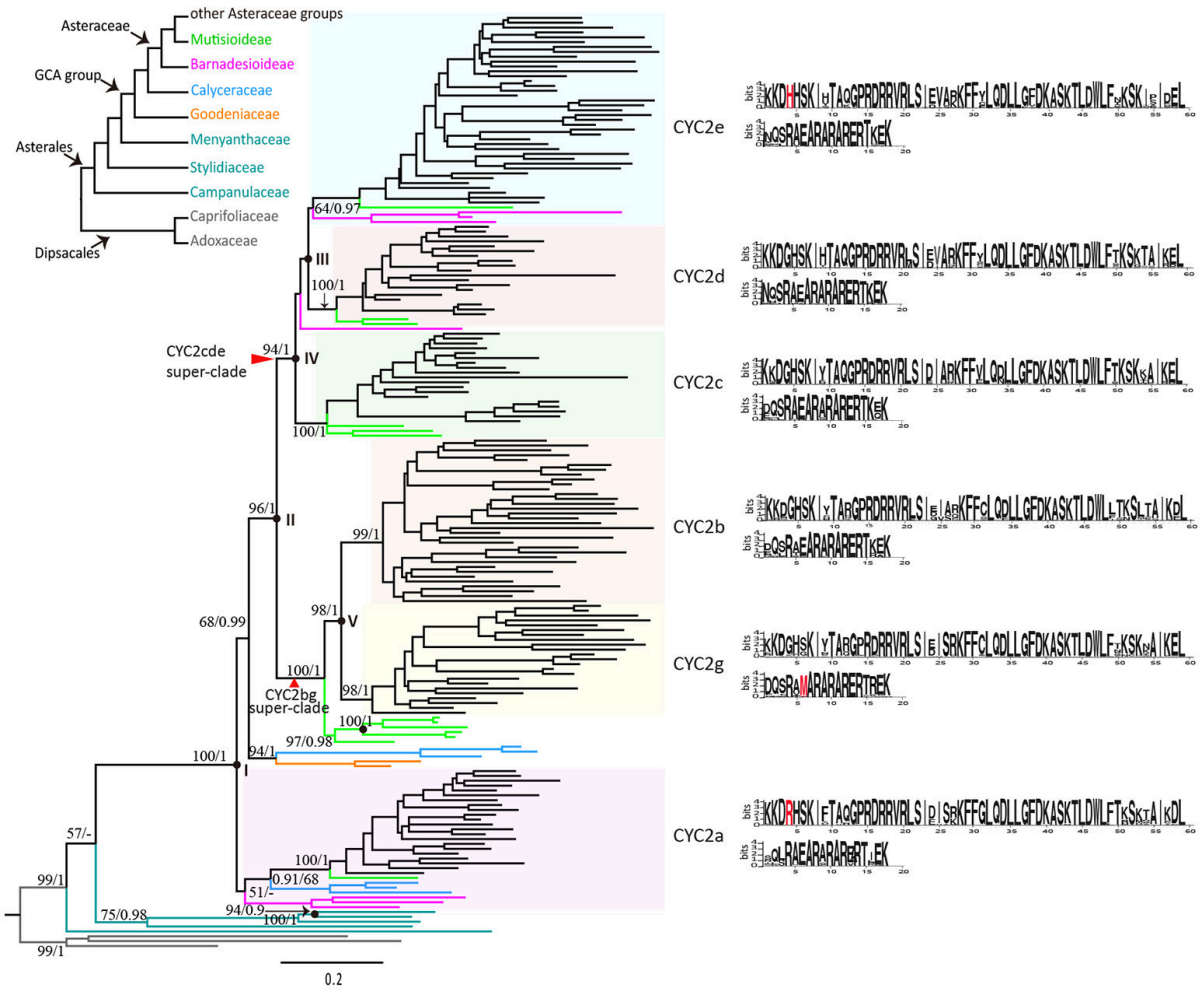
Relationships of *CYC2*-like genes in the Asterales based on 192 deduced amino acid sequences. The tree was constructed with Maximum Likelihood method (ML), using JTT+I+G+F model by1000 bootstrap replicates and rooted by sequences of Dipsacales as the outgroup. Bootstrap supports (by ML)/Posterior probabilities (by Bayesian Inference) are indicated above major branches. Black spots indicate gene duplication events; duplications within the Goodeniaceae-Calyceraceae-Asteraceae (GCA) group are labeled with roman letters I~V. Consensus sequences of TCP and R domains for each of the six CYC2 clades based on MEME test are shown in the right, and clade-specific amino acid fixations are in red.

In total 192 *CYC2*-like protein sequences were included in the phylogenetic analyses. Four gene trees constructed on different regions of the protein sequences are shown in Figure S2. The trees using TCP and R domains as well as using the region from TCP to R domains had lower resolution (Figures S2A,B) than the tree on the sequences from N-terminal to R domain (Figure S2C), and the full-length sequences resulted in even better resolved tree with relatively high supporting values (Figure S2D). The detailed differences between the four trees are described below. The tree based on the two conserved domains did not resolve the relationships among any *CYC2*-like copies (Figure S2A). When the less conserved sequences between the TCP and R domains were added, four clades were resolved with bootstrap supports above 50%: (i) the clade consisting of all sequences except the *CYC2*-like sequences of ancestral status, (ii) the clade consisting of all Asteraceae *CYC2*-like sequences except *CYC2a*, (iii) the CYC2bg clade (including a resolved CYC2b subclade), and (iv) the CYC2d clade (Figure S2B). Furthermore, the region from N-terminal to R domain resolved subclades CYC2c and CYC2g additionally (Figure S2C). Finally, the aligned full-length coding region could confirm six major copies of the Asteraceae *CYC2*-like genes (Figure S2D). Therefore, we tend to regard the duplication history of the Asteraceae *CYC2*-like genes as what the full-length sequences depicted (Figure 2).

The naming system of the *CYC2*-like genes of the present study basically follows Chapman et al. (2008) in sunflower, that is, five clades from CYC2a to CYC2e. The sixth clade was newly named as CYC2g by the present study (“f” was skipped because, Huang et al., 2016 reported a gene called *CmCYC2f* in *C. morifolium*, which is actually a *CYC2e*-like gene according to our study).

According to the gene tree (Figure 2), the six clades of *CYC2*-like genes can be traced back through five duplication events to a single ancestral copy that already existed in the common ancestor of the monophyletic Goodeniaceae-Calyceraceae-Asteraceae (GCA) group. The first duplication (Figure 2: I) occurred probably prior to the divergence of the GCA group, giving rise to two members, one of which has been maintained as the *CYC2a* gene in the extant Asteraceae and Calyceraceae families but probably lost in the Goodeniaceae family, while the other has retained in the extant Calyceraceae and Goodeniaceae but underwent a further duplication (Figure 2: II) in the ancestor of Asteraceae resulting in the split of the CYC2bg and the CYC2cde super-clades.

Soon after that, the CYC2cde super-clade went through two duplication events. One event (Figure 2: III) resulted in the CYC2e clade and a putative CYC2cd clade before the split of the basal-most subfamily Barnadesioideae, and the other event (Figure 2: IV) brought about CYC2c and CYC2d clades, predating the separation of Mutisioideae *sensu lato* (including Mutisioideae *sensu stricto* and Wunderlichioideae). The CYC2bg super-clade was divided into the CYC2b and CYC2g clades (Figure 2: V) between the split of Carduoideae and Mutisioideae. In addition, many specific replicates were found arising within different Asteraceae CYC2 clades (Table S6).

After the five duplications, some of the *CYC2*-like copies became dysfunctional or were lost in some lineages or capitulum types (Figures 1, 2 and Table S1). For example, *CYC2c*-like genes have not been detected in radiate and disciform species of the tribes Pertyeae, Arctotideae, Gnaphalieae, Astereae, Anthemideae and Inuleae; *CYC2g*-like genes were absent in Senecioneae and Helenieae; and *CYC2a*- and *CYC2d*-like genes were not isolated either by 3′-RACE PCR or amplification in genomic DNA of *Gnaphalium affine* (Gnaphalieae) despite of using numerous kinds of primer combinations (Table S2). In the genomic sequencing data of *L. sativa, CYC2d*-like gene was not found, and it was neither isolated from the five liguliflorous species of Cichorieae analyzed in this study. Nonetheless, *CYC2d*-like was identified in tribe Arctotideae which is sister to Cichorieae but has radiate capitula. In the ray-less tribe Cynareae, which has discoid capitula, transcripts of CYC2c, CYC2d, and CYC2g clade genes could not be detected (Figure 1 and Table S1). Additionally, the genomic data of *Cy. cardunculus* var. *scolymus* in this tribe suggested that *CYC2c* isoforms underwent pseudogenization and that both the *CYC2d* and *CYC2g* genes were entirely lost in its genome (Figure 1 and Figure S1).

Structures of the *CYC2*-like genes in the Asterales are rather uniform, with slight modifications, that is, most of them contain no more than one intron immediately before or after the stop codon. There were a few genes containing two introns, for instance, in *GriCYC2g* of *Gazania rigens* and *TpCYC2b* of *Tagetes patula*: the first intron was positioned before the stop codon, and the second was located in the 3′ UTR region (Figure S3).

MEME tests found fourteen common motifs (M1~M14) in the majority of the *CYC2*-like proteins of the GCA group (Figures S3, S4). M1 and M2 are two highly conserved motifs corresponding to the TCP and R domains respectively in the CYC/TB1 subfamily. M4 was located at the starting position of the CDS and was shared across all the proteins here. The rest of the motifs, except M9 and M10 that were completely absent in the *CYC2g* genes, were interspersed in the six CYC2 clade genes of the GCA group but lost randomly for some sequences in each of the clades. Clade-specific motifs are rare except two specific motifs were found in CYC2a and CYC2d clades, respectively (Figure S4). Notably, some characteristic amino acid sites were found in the TCP and R domains to discriminate *CYC2* members respectively. Of them, newly identified *CYC2g* shows the unique hydrophobic “M/V” replace the hydrophilic “E” in its R domain (Figure 2: highlighted in red).

### Expression patterns of *CYC2*-like genes in different capitulum types and different asteraceae lineages

**Figure 3:**
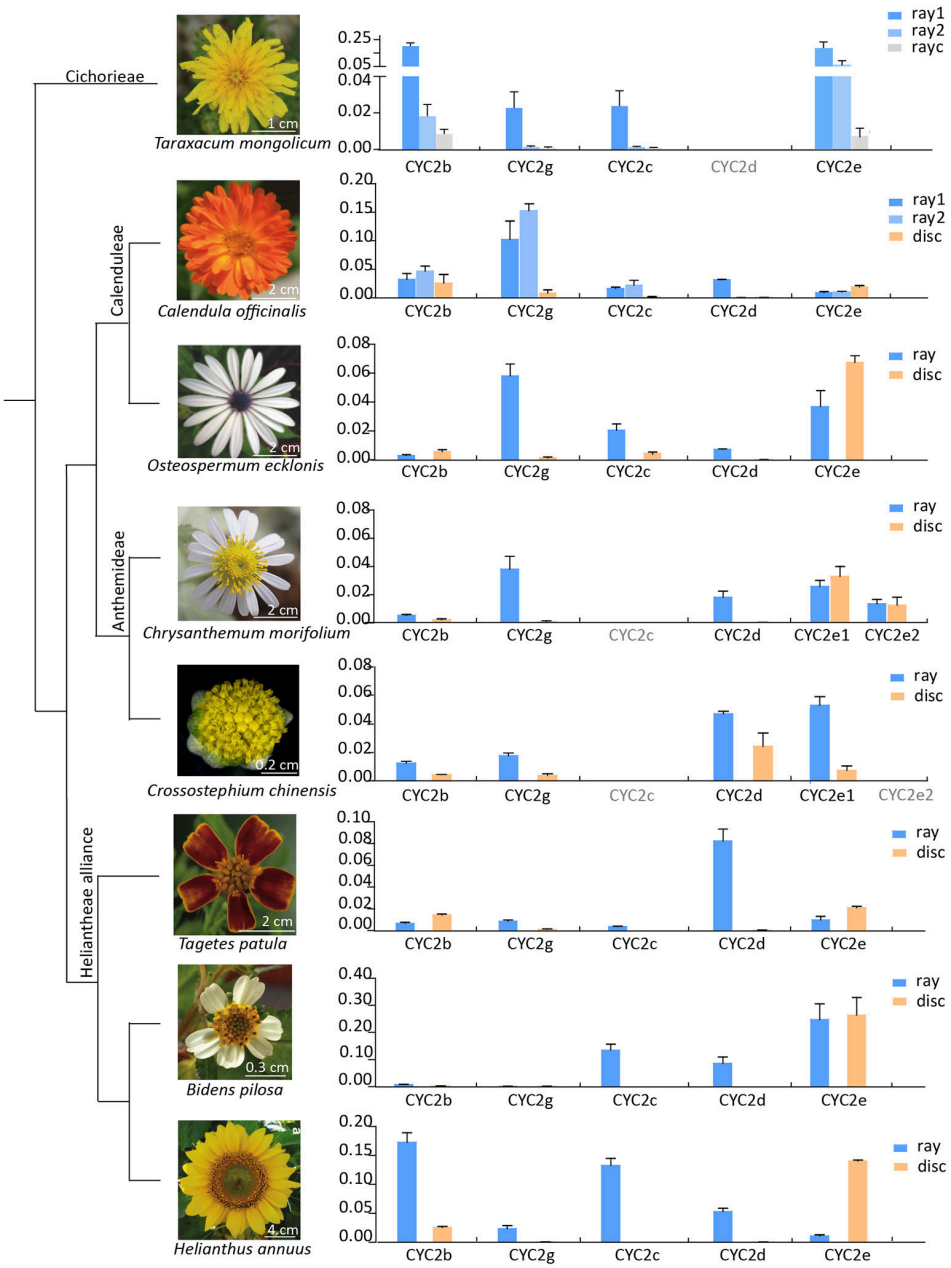
Relative expression levels of the Asteraceae *CYC2*-like genes except *CYC2a* in eight species that represent major lineages of the family (Panero and Funk, 2008) and major capitulum types (liguliflorous, radiate and disciform capitula). Genes with their names in gray was not found or lost in the species. Gene expression levels were normalized against expression levels of *ACTIN7*. Error bar is ± s.e. value of three biological samples. “ray1” and “ray2” for *Taraxacum mongolicum* and *Calendula officinalis* mean the first and second whole of rays, and “rayc” for *T. mongolicum* means central rays.

The expression patterns of *CYC2*-like genes (except *CYC2a*) in eight species representing liguliflorous, radiate and disciform capitula were analyzed (Figure 3). In the liguliflorous capitulum of *Taraxacum mongolicum* (Cichorieae), *CYC2b, 2c, 2e*, and *2g* genes presented decreasing expression levels from the outer to the inner florets. In the radiate species, *CYC2b* and *2e* were expressed in all florets, whereas, the expressions of *CYC2c, 2d*, and *2g* were limited in rays in most cases or showing a sudden decrease from the ray to the disc florets. *CYC2c* or *CYC2g* appeared to be ray-specific and were expressed alternatively in different Asteraceae lineages. For instance, in the ray florets of the Calenduleae-Gnaphalieae-Astereae-Anthemideae (CGAA) group, *CYC2g* was often highly expressed while *CYC2c* was weakly expressed or even lost. In contrast, in the ray florets of the Heliantheae alliance (HA), *CYC2c* was more often highly expressed while *CYC2g* was weak. In the disciform *Crossostephium chinensis* of the tribe Anthemideae, both *CYC2g* and *CYC2d* showed a higher expression in the marginal than in the central florets. However, such a expression difference is much bigger in its radiate relatives, e.g., in *Chrysanthemum morifolium*, expression of *CYC2g* and *CYC2d* is much higher in the marginal ray than in the central disc florets (Figure 3 and Figure S5).

**Figure 4:**
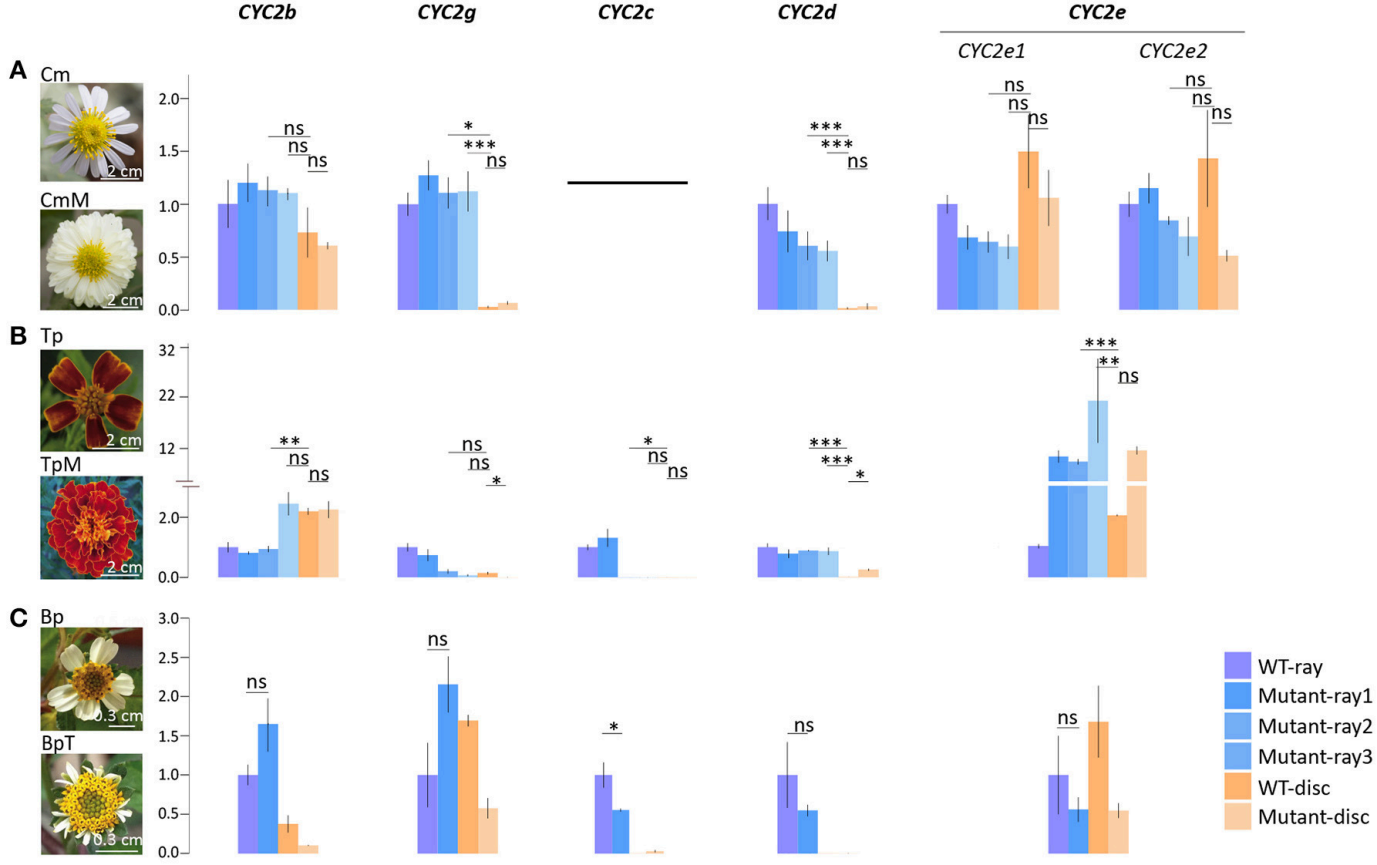
Comparative expression analysis of the Asteraceae *CYC2*-like genes in different types of capitulum. **(A)** Comparison between the wild type *Chrysanthemum morifolium* (Cm) and its multiradiata cultivar (CmM). **(B)** Comparison between the wild type *Tagetes patula* (Tp) and its multiradiata cultivar (TpM). **(C)** Comparison between the wild type *Bidens pilosa* (Bp) and its natural trans-ray mutant (BpT). Expression levels of *ACTIN7* were used for normalization; expression levels of each *CYC2* copy in the cultivar were normalized against its expression in the ray florets of the wild type via the 2^−ΔΔC_T_^ method (Livak and Schmittgen, 2001). Error bar is ± s.e. value of three biological samples. The two-tailed *t-*test was used for statistical tests: ^*^P ≤ 0.05; ^**^P ≤ 0.01; ^***^P ≤ 0.001.

To further verify whether *CYC2c*-like and *CYC2g*-like genes predominately or independently determine the differentiation between ray and disc florets in different lineages, qPCR analyses were conducted on three infra-species pairs of varied phenotypes, i.e., *C*. *morifolium* wild-type/multiradiata cultivar (with multiwhorl ray florets), *T. patula* wild-type/multiradiata cultivar, and *Bidens pilosa* wild-type/trans-ray mutant (with visible dorsal petals and a fertile stamen in marginal ray-like florets) (Figure 4). In the *C. morifolium* mutant (CmM), the expression of the ray-specific *CmCYC2g* and *2d* genes extended to the inner whorls of florets (Figure 4A). Similar expansions of *TpCYC2c* and *2d* expression were found in the *T. patula* mutant (TpM), but *TpCYC2g* did not show significant expression expansion (Figure 4B). In the *B. pilosa* mutant (BpT), downregulation of *BpCYC2c* and *2d* appeared to be linked, although the change was not statistically significant for *BpCYC2d* (Figure 4C).

### Overexpression of the *CLCYC2d* gene in *Chrysanthemum lavandulifolium*

**Figure 5:**
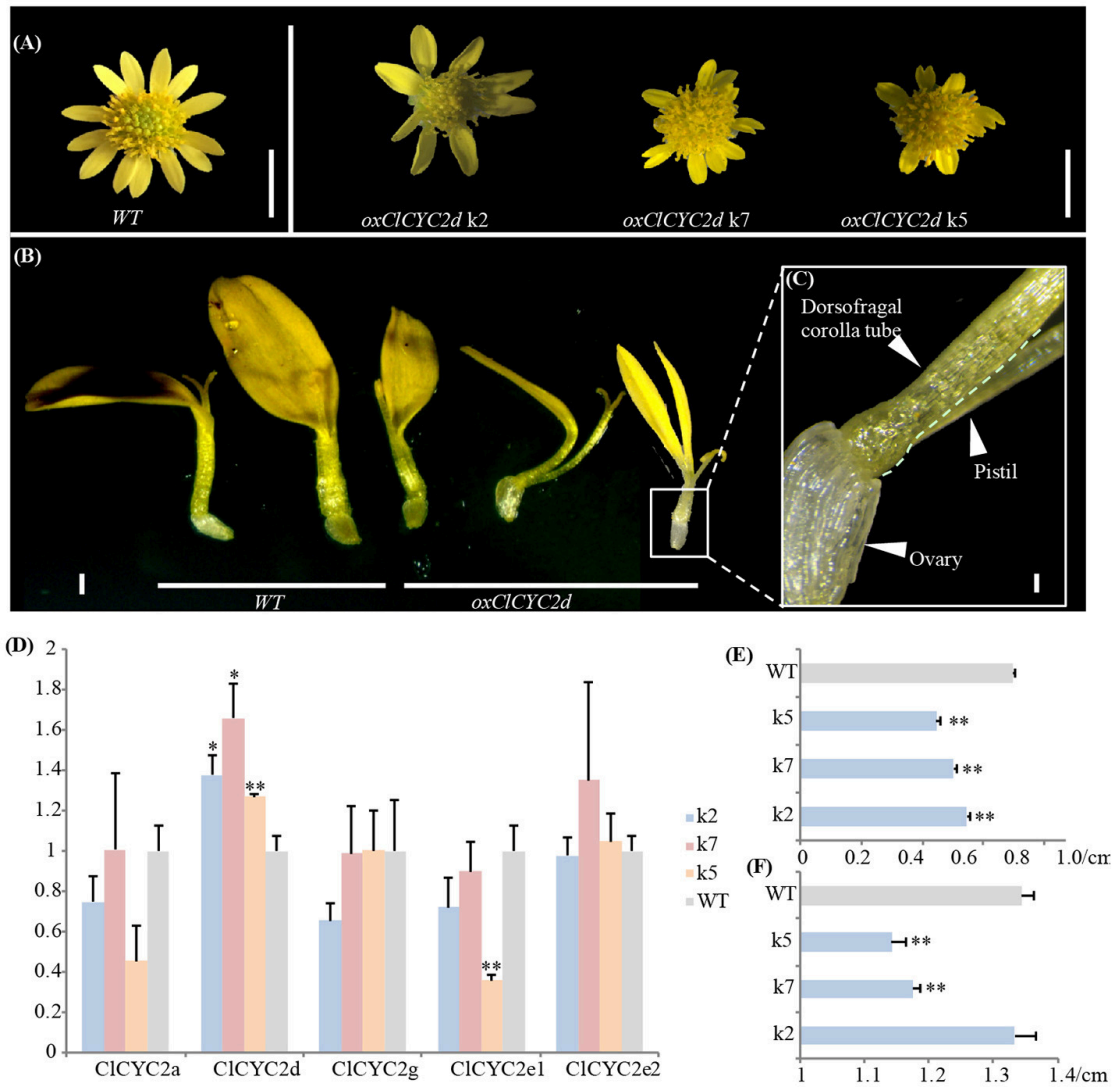
Ectopic expression of *ClCYC2d* of *Chrysanthemum lavandulifolium* suppresses the growth of rays. **(A–C)** Capitulum and ray phenotypes of transgenic lines compared to wild-type plants. Bars = 5 mm **(A)**, 1 mm **(B)**, and 0.1 mm **(C)**. **(D)** Changes in expression of *CYC2*-like genes between transgenic lines and the wildtype. **(E,F)** Changes in lengths of ray flowers and diameters of capitula. Error bar is ± s.e. value of three biological samples. Statistical significance were tested using the two-tailed *t*-test: ^*^P ≤ 0.05; ^**^P ≤ 0.01.

We noticed that constitutive expression of *ClCYC2d* distinctly repressed the growth of corolla during ray development in three transgenic lines. The positive transgenic lines were verified (Figure S6). The ventral ligule became shorter and deeply split, and the corolla tube split completely at the adaxial side of some ray florets (Figures 5A–C). The lengths of all the ray florets and diameters of the capitula clearly differed from those of the wild type based on precise calculations for each of the transgenic lines (Figures 5E,F). Correspondingly, the transcription level of *ClCYC2d* increased significantly in the blooming capitulum (at the stage when the outmost layer of the disc florets opens) after transformation; the expression of other *CYC2* copies changed insignificantly (Figure 5D).

## Discussion

### The complexity of the capitulum architecture is related to the increase in the *CYC2*-like gene members in the asteraceae

To explore whether the determination of floral zygomorphy in the radiate capitulum is pervasively lineage independent, our phylogenetic analysis, which was based on a broad taxon sampling and full-length sequencing of the coding region of the *CYC2*-like genes in the Asteraceae and its allied families of the Asterales, demonstrates that five duplication events have generated six major *CYC2* copies in Asteraceae since the ancestor of the monophyletic GCA group (Figure 2). In a previous study, two *CYC2*-like genes, *AtCYC2a1* and *AtCYC2a2*, were isolated from *Acicarpha tribuloides* (Calyceraceaee) and were regarded as lineage-specific *CYC2a* duplicates (Chapman et al., 2012). Our analysis proves that the orthologs of *AtCYC2a1* indeed belong to the CYC2a clade genes, while *AtCYC2a2* is actually orthologous to an ancestral copy that generated all the other *CYC2* copies within Asteraceae (Figure 2 and Figure S7).

In the basal subfamily Barnadesioideae of Asteraceae, Chapman et al. (2012) isolated three *CYC2*-like genes from *Dasyphyllum diacanthoides*, regarded them the CYC2a and CYC2c clade genes and suggested that genes of the CYC2b, CYC2d and CYC2e clades might be lost in that species. The present phylogenetic analysis showed that the *CYC2*-like genes we isolated from the Barnadesioideae plants were orthologous to the three *DdCYC2*-like genes (Figure S7), but were assigned to the CYC2a and CYC2e clades and an undifferentiated CYC2cd member (Figure 2). In addition to the CYC2a~2e clades documented previously (Chapman et al., 2008, 2012; Kim et al., 2008; Tähtiharju et al., 2012; Garcês et al., 2016), we authenticated and characterized an additional CYC2g clade that is sister to the CYC2b clade. The ancestral gene before the split of CYC2b and CYC2g clades was probably lost in the basal subfamily Barnadesioideae but underwent specific duplication within Mutisioideae *sensus lato* (Figure 2).

All in all, five duplication events that started from a single-copy gene in the common ancestor of the Goodeniaceae-Calyceraceae-Asteraceae group and ended before the divergence of the subfamily Carduoideae led to six CYC2 clade genes in the Asteraceae. In addition, lineage-specific duplications occurred frequently within each of the CYC2 clades. To tease apart relationships of the *CYC2*-like genes, we tried to clarify discrepancies in their functions previously found in distantly related Asteraceae species.

### Changes in capitulum types and floret morphs may have resulted from the option and interaction of different *CYC2-*like gene copies

Previous studies have shown diverse expression patterns and the parallel recruitment of *CYC2-*like genes in the determination and modification of zygomorphic ray florets across distantly related Asteraceae lineages/taxa (Broholm et al., 2008; Chapman et al., 2008, 2012; Kim et al., 2008; Juntheikki-Palovaara et al., 2014; Garcês et al., 2016; Huang et al., 2016). Through investigations with various florets and capitula as materials, we find each *CYC2-*like member generally has a constant spatial expression pattern throughout the family (Figure 3), e.g., *CYC2c, 2g* and *2d* are expressed prominently in ray florets of the radiate capitulum (Figure 3; Chapman et al., 2008; Tähtiharju et al., 2012). In the discoid capitula of Carduoideae, *CYC2c, 2g*, and *2d* all show dysfunction (Figure S1), suggesting they be necessary for the ray development. Further evidence of the role of *CYC2d* in the development of a ray floret can be found in the tribe Arctotideae which carries radiate capitula and its sister group, tribe Cichorieae, which has liguliflorous capitula of bisexual ligulate florets. *CYC2d* is present in the radiated Arctotideae but is absent in transcripts and the genomic data of Cichorieae (Figure S1 and Table S1). This indicates the loss of *CYC2d* may be related to the recovery of dorsal petals and stamens of the bisexual ligulate florets of Cichorieae from a ray floret of the closely related taxa. Experimental data of overexpressing *CYC2d* orthologs in *C*. *lavandulifolium* (Figure 5) and *S. vulgaris* (Kim et al., 2008) further confirmed *CYC2d* as repressors of the development of floral organs of rays.

Different from *CYC2d*, which is restricted to modulating the morphology of rays, *CYC2c* and *2g* have been proved to promote morphogenesis of ray florets in *H. annuus* and *C. morifolium*, respectively (Chapman et al., 2012; Huang et al., 2016). Our comparative analysis of the multiradiata mutants in the CGAA and HA groups supports the findings above (Figures 4A,B). The degeneration of ray characteristics is found to be highly associated with a reduction in *CYC2c* expression in *H. annuus* (Fambrini et al., 2011; Chapman et al., 2012) and *B*. *pilosa* (Figure 4C). Similarly, in the disciform *Crossotephium chinensis* of the tribe Anthemideae, a decrease in *CYC2g* expression in the marginal female florets to a level nearly as low as that in the disc florets may account for its separate petal lobes instead of a fused ligule (Figure 3 and Figure S5). Interestingly, the role of either *CYC2c* or *2g* in determining ray identity seems optional, and this alteration appears iteratively since the divergence of the subfamily Pertyoideae (Figures 1, 4).

Our experimental data revealed co-expression pattern between *CYC2c*/*2g* and *CYC2d* (Figure 4), suggesting they cooperate in the CYC2 regulatory framework to determine the transfer between rays and discs. Yang et al. (2012) reported the autoregulatory cycle between two duplicated *CYC2*-like genes first in *Primulina heterotricha* (Gesneriaceae).

Transgenic phenotypes by reducing the expression of *GhCYC2* (a *CYC2e* member) in *G. hybrida* (Broholm et al., 2008) and *RAY3* (a *CYC2b* member) in *S*. *vulgaris* (Garcês et al., 2016) implicated a potential effect of *CYC2b* and *2e* on the ligule growth of ray florets. In addition, we found loss or malfunction of *CYC2e* and *2b* in *L. sativa* and *C. lavandulifolium*, respectively (Figures 1, 5; Table S1), suggesting those two genes have redundant function and work independently in different tribes.

### Capitulum evolution is highly linked to the development of floral heteromorphism regulated by the ray development program

The complexity and flexibility of modular programs play a crucial role in character innovations, and this is often correlated with the divergence of key regulators (Moyroud and Glover, 2017). According to the findings of previous and present studies, the complicated regulatory network of *CYC2*-like genes in the Asteraceae originated from an ancestral homolog within the Asterales, and these genes have been co-opted, relying on functional specificity and internal regulation after their duplication. The roles of those Asteraceae *CYC2*-like genes, except *CYC2a*, can be interpreted as follows:

Within the ray development program, *CYC2c* and *2g* determine the position and zygomorphy of ray florets (this study; Chapman et al., 2012; Huang et al., 2016); *CYC2d* represses the growth of stamens and dorsal corolla lobes (this study; Kim et al., 2008); *CYC2e* and *2b* regulate the ligule length of ray florets (Broholm et al., 2008; Garcês et al., 2016). The elongation of the ventral ligule of the ray florets are also proven to involve *MYB*-like genes (*SvDIV1B* and *SvRAD*) (Garcês et al., 2016). In *SvDIV1B*-RNAi lines of *S*. *vulgaris, RAY3*, and *SvRAD* are distinctly up-regulated, but not vice versa (Garcês et al., 2016). The antagonistic inter-regulation between the *CYC2*-like and *DIV*-like genes in the Asteraceae appears as an inverse case to the regulation mode observed in *Antirrhinum majus* (Corley et al., 2005; Costa et al., 2005). So far, all the experimental data suggest that *CYC2*-like genes are involved in both the dorsal and ventral morphogenesis of the Asteraceae ray flowers, which differs from their dorsal-specific regulations of zygomorphic flowers in *A. majus* and other core eudicots (Feng et al., 2006; Zhang et al., 2010; Howarth et al., 2011; Busch et al., 2012; Zhong and Kellogg, 2014).

**Figure 6:**
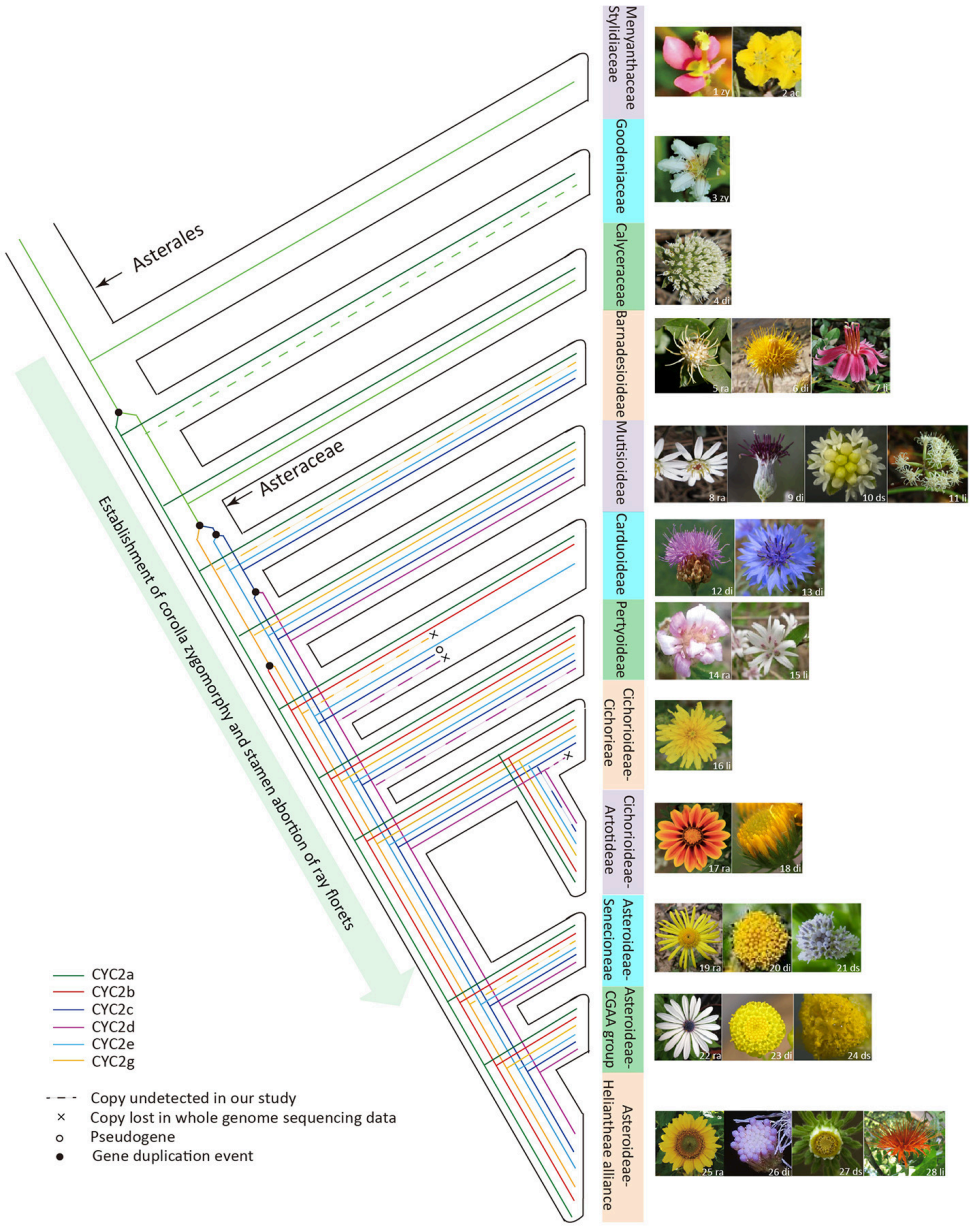
The hypothesized evolutionary trajectory of the Asteraceae capitulum architecture in relation to the evolution of the *CYC2*-like genes. The phylogeny framework of Asterales is depicted according to Panero and Funk (2008), Funk et al. (2009) and APG IV (Chase et al., 2016). Images of the typical flower or inflorescence/capitulum are attached to taxon branches. Abbreviations on the floral images denote the floral symmetry or capitulum type: zy, zygomorphy; ac, actinomorphy; ra, radiate; di, discoid; li, liguliflorous; ds, disciform. Sources of flora images are indicated by the numbers on the photos: 1, 2, 8, 10, 14–17, 22–25, and 27 were photographed by the present authors; 9 is cited from Panero et al. (2014); 3–4, 6, 11, and 28 are from Wikipedia (https://www.wikipedia.org/); 5, 18 and 21 from the website https://www.flickr.com/photos; 7 from http://flickriver.com/photos/grandma-shirley/7181225103/; 12 from http://www.cd-pa.com; 13 from http://www.naturspaziergang.de; 19 from http://luirig.altervista.org; 20 from https://image.baidu.com; 26 from http://publish.plantnet-project.org.

In sum, based on the present phylogenetic and expression analyses of the Asteraceae *CYC2*-like genes, we may conclude that an increase in the copy number of *CYC2*-like genes allows for the increase of capitulum morphological variation. Some of the *CYC2*-like genes trigger the floral heteromorphism and all of them partake in regulating allometric growth and fusion of corolla lobes, and stamen regression. The complex pattern of capitulum evolution is mainly characterized by iterative transitions among radiate, discoid, disciform and ligulate types, which is obviously associated with the functional divergence as well as the gain/loss of ray-specific *CYC2*-like members (Figure 6). Moreover, transitions among divergent capitula may be under the selective constraint of pollinators. It is likely that the evolutionary radiation of Asteraceae was driven, at least in part, by the adaptive transitions among different capitula. Therefore, the evolution of the biggest plant family is certainly associated with the expansion and evolution of the *CYC2*-like genes.

## Author contributions

G-YR and Y-PG conceived and directed the research. JC and C-ZS designed and performed experiments, and analyzed data. JC and Y-PG wrote the manuscript. C-ZS and G-YR helped on manuscript writing.

### Conflict of interest statement

The authors declare that the research was conducted in the absence of any commercial or financial relationships that could be construed as a potential conflict of interest. The reviewer Y-ZW and handling Editor declared their shared affiliation.

## References

[B1] Andersson S. (1999). The cost of floral attractants in *Achillea ptarmica* (Asteraceae): evidence from a ray removal experiment. Plant Biol. 1, 569–572. 10.1111/j.1438-8677.1999.tb00785.x

[B2] Andersson S. Widén B. (1993). Pollinator-mediated selection on floral traits in a synthetic population of *Senecio integrifolius* (Asteraceae). Oikos 66, 72–79. 10.2307/3545197

[B3] Badouin H. Gouzy J. Grassa C. J. Murat F. Staton S. E. Cottret L.. (2017). The sunflower genome provides insights into oil metabolism, flowering and Asterid evolution. Nature 546, 148–152. 10.1038/nature22380 28538728

[B4] Bailey T. L. Boden M. Buske F. A. Frith M. Grant C. E. Clementi L.. (2009). MEME SUITE: tools for motif discovery and searching. Nucleic Acids Res. 37, W202–W208. 10.1093/nar/gkp335 19458158 PMC2703892

[B5] Balavoine G. (1996). Identification of members of several homeobox genes in a planarian using a ligation-mediated polymerase chain reaction technique. Nucleic Acids Res. 24, 1547–1553. 10.1093/nar/24.8.1547 8628690 PMC145808

[B6] Bello M. A. Cubas P. Álvarez I. Sanjuanbenito G. Fuertes-Aguilar J. (2017). Evolution and expression patterns of *CYC/TB1* genes in *Anacyclus*: phylogenetic insights for floral symmetry genes in Asteraceae. Front. Plant Sci. 8:589. 10.3389/fpls.2017.00589 28487706 PMC5403951

[B7] Bremer K. (1994). Asteraceae: Cladistics and Classification. Portland, OR: Timber Press.

[B8] Broholm S. K. Tähtiharju S. Laitinen R. A. Albert V. A. Teeri T. H. Elomaa P. (2008). A TCP domain transcription factor controls flower type specification along the radial axis of the *Gerbera* (Asteraceae) inflorescence. Proc. Natl. Acad. Sci. U.S.A. 105, 9117–9122. 10.1073/pnas.0801359105 18574149 PMC2449374

[B9] Busch A. Horn S. Mühlhausen A. Mummenhoff K. Zachgo S. (2012). Corolla Monosymmetry: evolution of a morphological novelty in the Brassicaceae Family. Mol. Biol. Evol. 29, 1241–1254. 10.1093/molbev/msr297 22135189

[B10] Chapman M. A. Abbott R. J. (2010). Introgression of fitness genes across a ploidy barrier. New Phyto. 186, 63–71. 10.1111/j.1469-8137.2009.03091.x 19912548

[B11] Chapman M. A. Leebens-Mack J. H. Burke J. M. (2008). Positive selection and expression divergence following gene duplication in the sunflower *CYCLOIDEA* gene family. Mol. Biol. Evol. 25, 1260–1273. 10.1093/molbev/msn001 18390478

[B12] Chapman M. A. Tang S. Draeger D. Nambeesan S. Shaffer H. Barb J. G.. (2012). Genetic analysis of floral symmetry in Van Gogh's sunflowers reveals independent recruitment of *Cycloidea* genes in the Asteraceae. PLoS Genet. 8:e1002628. 10.1371/journal.pgen.1002628 22479210 PMC3315478

[B13] Chase M. W. Christenhusz M. J. M. Fay M. F. Byng J. W. Judd W. S. Soltis D. E. (2016). An update of the Angiosperm Phylogeny Group classification for the orders and families of flowering plants: APG IV. Bot. J. Linn. Soc. 181, 1–20. 10.1111/boj.12385

[B14] Classen-Bockhoff R. (1990). Pattern analysis in pseudanthia. Plant Sys. Evol. 171, 57–88. 10.1007/BF00940596

[B15] Coen E. S. Nugent J. M. (1994). Evolution of flowers and inflorescences. Development 1994 (suppl.), 107–116.

[B16] Corley S. B. Carpenter R. Copsey L. Coen E. (2005). Floral asymmetry involves an interplay between TCP and MYB transcription factors in *Antirrhinum*. Proc. Natl. Acad. Sci. U.S.A. 102, 5068–5073. 10.1073/pnas.0501340102 15790677 PMC555980

[B17] Costa M. M. Fox S. Hanna A. I. Baxter C. Coen E. (2005). Evolution of regulatory interactions controlling floral asymmetry. Development 132, 5093–5101. 10.1242/dev.02085 16236768

[B18] Darriba D. Taboada G. L. Doallo R. Posada D. (2011). ProtTest 3: fast selection of best-fit models of protein evolution. Bioinformatics 27, 1164–1165. 10.1093/bioinformatics/btr088 21335321 PMC5215816

[B19] Endress P. K. (2011). Evolutionary diversification of the flowers in angiosperms. Am. J. Bot. 98, 370–396. 10.3732/ajb.1000299 21613132

[B20] Fambrini M. Bertini D. Pugliesi C. (2003). The genetic basis of a mutation that alters the floral symmetry in sunflower. Ann. Appl. Biol. 143, 341–347. 10.1111/j.1744-7348.2003.tb00303.x

[B21] Fambrini M. Salvini M. Pugliesi C. (2011). A transposon-mediate inactivation of a *Cycloidea*-like gene originates polysymmetric and androgynous ray flowers in *Helianthus annuus*. Genetica 139, 1521–1529. 10.1007/s10709-012-9652-y 22552535

[B22] Feng X. Zhao Z. Tian Z. Xu S. Luo Y. Cai Z.. (2006). Control of petal shape and floral zygomorphy in *Lotus japonicus*. Proc. Natl. Acad. Sci. U.S.A. 103, 4970–4975. 10.1073/pnas.0600681103 16549774 PMC1458779

[B23] Ford V. Gottlieb L. (1990). Genetic studies of floral evolution in *Layia*. Heredity (Edinb). 64, 29–44. 10.1038/hdy.1990.5

[B24] Funk V. A. Susanna A. Stuessy T. F. Bayer R. J. (2009). Systematics, Evolution, and Biogeography of Compositae. Vienna: IAPT.

[B25] Garcês H. M. P. Spencer V. M. Kim M. (2016). Control of floret symmetry by *RAY3, SvDIV1B* and *SvRAD* in the capitulum of *Senecio vulgaris*. Plant Physiol. 17, 2055–2068. 10.1104/pp.16.00395 PMC4936572 27208229

[B26] Gillies A. C. M. Cubas P. Coen E. S. Abbott R. J. (2002). Making rays in the Asteraceae: genetics and evolution of radiate versus discoid flower heads, in Developmental Genetics and Plant Evolution, eds. Cronk Q. C. B. Bateman R. M. Hawkins J. A. (London: Taylor & Francis Group), 233–246.

[B27] Hall T. A. (1999). BioEdit: a user-friendly biological sequence alignment editor and analysis program for Windows 95/98/NT. Nucleic Acids Symp. Ser. 41, 95–98.

[B28] Harder L. D. Prusinkiewicz P. (2013). The interplay between inflorescence development and function as the crucible of architectural diversity. Ann. Bot. 112, 1477–1493. 10.1093/aob/mcs252 23243190 PMC3828939

[B29] Harris E. M. (1995). Inflorescence and floral ontogeny in Asteraceae: a synthesis of historical and current concepts. Bot. Rev. 61, 93–278. 10.1007/BF02887192

[B30] Harris E. M. (1999). Capitula in the Asteridae: a widespread and varied phenomenon. Bot. Rev. 65, 348–366. 10.1007/BF02857754

[B31] Hileman L. C. (2014). Bilateral flower symmetry—how, when and why? Curr. Opin. Plant Biol. 17, 146–152. 10.1016/j.pbi.2013.12.002 24507506

[B32] Howarth D. G. Martins T. Chimney E. Donoghue M. J. (2011). Diversification of *Cycloidea* expression in the evolution of bilateral flower symmetry in Caprifoliaceae and *Lonicera* (Dipsacales). Ann. Bot. 107, 1521–1532. 10.1093/aob/mcr049 21478175 PMC3108805

[B33] Huang D. Sun M. Cheng T. R. Wang J. Zhang Q. X. (2016). Identification and characterization of *CYC*-like genes in regulation of ray flower development in *Chrysanthemum morifolium*. Front. Plant Sci. 7:1633 10.3389/fpls.2016.01633 27872631 PMC5097909

[B34] Hull P. (1974). Self-fertilisation and the distribution of the radiate form of *Senecio vulgaris* L. in central Scotland. Watsonia 10, 69–75.

[B35] Jeffrey C. (1977). Corolla forms in Compositae—some evolutionary and taxonomic speculations, in The Biology and Chemistry of the Compositae, eds. Heywood V. H. Harborne J. B. Turner B. L. (London: Academic Press), 111–118.

[B36] Jeffrey C. (2009). Evolution of Compositae flowers, in: Systematics, Evolution, and Biogeography of Compositae, eds. Funk V. A. Susanna A. Stuessy T. F. Bayer R. J. (Vienna: IAPT), 131–138.

[B37] Juntheikki-Palovaara I. Tähtiharju S. Lan T. Broholm S. K. Rijpkema A. S. Ruonala R.. (2014). Functional diversification of duplicated CYC2 clade genes in regulation of inflorescence development in *Gerbera hybrida* (Asteraceae). Plant J. 79, 783–796. 10.1111/tpj.12583 24923429

[B38] Kalisz S. Ree R. H. Sargent R. D. (2006). Linking floral symmetry genes to breeding system evolution. Trends Plant Sci. 11, 568–573. 10.1016/j.tplants.2006.10.005 17097332

[B39] Kim M. Cui M. L. Cubas P. Gillies A. Lee K. Chapman M. A.. (2008). Regulatory genes control a key morphological and ecological trait transferred between species. Science 322, 1116–1119. 10.1126/science.1164371 19008450

[B40] Kirchoff B. K. Claßen-Bockhoff R. (2013). Inflorescences: concepts, function, development and evolution. Ann. Bot. 112, 1471–1476. 10.1093/aob/mct267 24383103 PMC3828949

[B41] Ledger S. E. Deroles S. C. Given N. K. (1991). Regeneration and *Agrobacterium*-mediated transformation of chrysanthemum. Plant Cell Rep. 10, 195–199. 10.1007/BF00234294 24221545

[B42] Leppik E. E. (1977). The evolution of capitulum types of the Compositae in the light of insect-flower interaction, in The Biology and Chemistry of the Compositae, eds. Heywood V. H. Harborne J. B. Turner B. L. (London: Academic Press), 61–89.

[B43] Livak K. J. Schmittgen T. D. (2001). Analysis of relative gene expression data using real-time quantitative PCR and the 2^−ΔΔC_T_^ method. Methods 25, 402–408. 10.1006/meth.2001.1262 11846609

[B44] Löytynoja A. Goldman N. (2010). webPRANK: a phylogeny-aware multiple sequence aligner with interactive alignment browser. BMC Bioinformatics 11:579. 10.1186/1471-2105-11-579 21110866 PMC3009689

[B45] Luo D. Carpenter R. Copsey L. Vincent C. Clark J. Coen E. (1999). Control of organ asymmetry in flowers of *Antirrhinum*. Cell 99, 367–376. 10.1016/S0092-8674(00)81523-8 10571179

[B46] Luo D. Carpenter R. Vincent C. Copsey L. Coen E. S. (1996). Origin of floral asymmetry in *Antirrhinum*. Nature 383, 794–799. 10.1038/383794a0 8893002

[B47] Mandel J. R. Dikow R. B. Funk V. A. (2015). Using phylogenomics to resolve mega-families: an example from Compositae. J. Syst. Evol. 53, 391–402. 10.1111/jse.12167

[B48] Mitiouchkina T. Y. Dolgov S. V. (2000). Modification of chrysanthemum flower and plant architecture by *rolC* gene from *Agrobacterium rhizogenes* introduction. Acta Hortic. 508, 163–172. 10.17660/ActaHortic.2000.508.21

[B49] Moyroud E. Glover B. J. (2017). The Evolution of Diverse Floral Morphologies. Curr. Biol. 27, R941–R951. 10.1016/j.cub.2017.06.053 28898667

[B50] Nielsen L. R. Philipp M. Siegismund H. R. (2002). Selective advantage of ray florets in *Scalesia affinis* and *S. pedunculata* (*Asteraceae*), two endemic species from the Galápagos. Evol. Eco. 16, 139–153. 10.1023/A:1016301027929

[B51] Panero J. L. Freire S. E. Ariza Espinar L. Crozier B. S. Barbaza G. E. Cantero J. J. (2014). Resolution of deep nodes yields an improved backbone phylogeny and a new basal lineage to study early evolution of Asteraceae. Mol. Phylogenet. Evol. 80, 43–54. 10.1016/j.ympev.2014.07.012 25083940

[B52] Panero J. L. Funk V. A. (2002). Toward a phylogenetic subfamilial classification for the Compositae (Asteraceae). Proc. Biol. Soc. Wash. 115, 909–922.

[B53] Panero J. L. Funk V. A. (2008). The value of sampling anomalous taxa in phylogenetic studies: major clades of the Asteraceae revealed. Mol. Phylogenet. Evol. 47, 757–782. 10.1016/j.ympev.2008.02.011 18375151

[B54] Peng Y. Lai Z. Lane T. Nageswara-Rao M. Okada M. Jasieniuk M.. (2014). De novo genome assembly of the economically important weed horseweed using integrated data from multiple sequencing platforms. Plant Physiol. 166, 1241–1254. 10.1104/pp.114.247668 25209985 PMC4226366

[B55] Ren J. B. Guo Y. P. (2015). Behind the diversity: ontogenies of radiate, disciform, and discoid capitula of *Chrysanthemum* and its allies. J. Syst. Evol. 53, 520–528. 10.1111/jse.12154

[B56] Reuther K. Claßen-Bockhoff R. (2013). Andromonoecy and developmental plasticity in *Chaerophyllum bulbosum* (Apiaceae–Apioideae). Ann. Bot. 112, 1495–1503. 10.1093/aob/mct073 23585495 PMC3828945

[B57] Reyes-Chin-Wo S. Wang Z. Yang X. Kozik A. Arikit S. Song C.. (2017). Genome assembly with *in vitro* proximity ligation data and whole-genome triplication in lettuce. Nat. Commun. 8:14953. 10.1038/ncomms14953 28401891 PMC5394340

[B58] Ronquist F. Huelsenbeck J. P. (2003). MrBayes 3: bayesian phylogenetic inference under mixed models. Bioinformatics 19, 1572–1574. 10.1093/bioinformatics/btg180 12912839

[B59] Scaglione D. Reyes-Chin-Wo S. Acquadro A. Froenicke L. Portis E. Beitel C. (2016). The genome sequence of the outbreeding globe artichoke constructed *de novo* incorporating a phase-aware low-pass sequencing strategy of F1 progeny. Sci. Rep. 6:19427 10.1038/srep19427 26786968 PMC4726258

[B60] Spena A. Schmülling T. Koncz C. Schell J. (1987). Independent and synergistic activity of rol A, B and C loci in stimulating abnormal growth in plants. EMBO J. 6, 3891–3899. 16453816 10.1002/j.1460-2075.1987.tb02729.x PMC553866

[B61] Stamatakis A. (2014). RAxML version 8: a tool for phylogenetic analysis and post-analysis of large phylogenies. Bioinformatics 30, 1312–1313. 10.1093/bioinformatics/btu033 24451623 PMC3998144

[B62] Stuessy T. F. Spooner D. M. Evans K. A. (1986). Adaptive significance of ray corollas in *Helianthus grosseserratus* (Compositae). Am. Midl. Nat. 115, 191–197. 10.2307/2425849

[B63] Tähtiharju S. Rijpkema A. S. Vetterli A. Albert V. A. Teeri T. H. Elomaa P. (2012). Evolution and diversification of the CYC/TB1 gene family in Asteraceae—a comparative study in gerbera (Mutisieae) and sunflower (Heliantheae). Mol. Biol. Evol. 29, 1155–1166. 10.1093/molbev/msr283 22101417

[B64] Teo Z. W. N. Song S. Wang Y.-Q. Liu J. Yu H. (2014). New insights into the regulation of inflorescence architecture. Trends Plant Sci. 19, 158–165. 10.1016/j.tplants.2013.11.001 24315403

[B65] Trow A. (1912). On the inheritance of certain characters in the common groundsel—*Senecio vulgaris* Linn.—and its segregates. J. Genet. 2, 239–276. 10.1007/BF02981542

[B66] Ushimaru A. Dohzono I. Takami Y. Hyodo F. (2009). Flower orientation enhances pollen transfer in bilaterally symmetrical flowers. Oecologia 160, 667–674. 10.1007/s00442-009-1334-9 19333624

[B67] Wyatt R. (1982). Inflorescence architecture: how flower number, arrangement, and phenology affect pollination and fruit-set. Am. J. Bot. 69, 585–594. 10.1002/j.1537-2197.1982.tb13295.x

[B68] Yang X. Pang H. B. Liu B. L. Qiu Z. J. Gao Q. Wei L.. (2012). Evolution of double positive autoregulatory feedback loops in CYCLOIDEA2 Clade Genes is associated with the origin of floral zygomorphy. Plant Cell 24, 1834–1847. 10.1105/tpc.112.099457 22649271 PMC3442572

[B69] Zhang W. Kramer E. M. Davis C. C. (2010). Floral symmetry genes and the origin and maintenance of zygomorphy in a plant-pollinator mutualism. Proc. Natl. Acad. Sci. U.S.A. 107, 6388–6393. 10.1073/pnas.0910155107 20363959 PMC2851953

[B70] Zhao Y. Zhang T. Broholm S. K. Tähtiharju S. Mouhu K. Albert V. (2016). Co-opting floral meristem identity genes for patterning of the flower-like Asteraceae inflorescence. Plant Physiol. 172, 284–296. 10.1104/pp.16.00779 27382139 PMC5074612

[B71] Zhong J. Kellogg E. A. (2014). Duplication and expression of *CYC2*-like genes in the origin and maintenance of corolla zygomorphy in Lamiales. New Phytol. 205, 852–868. 10.1111/nph.13104 25329857

