# Supplementary material for: Patterning the Asteraceae Capitulum: Duplications and Differential Expression of the Flower Symmetry CYC2-Like Genes
Source: Front Plant Sci. 2018 Apr 25;9:551. doi: 10.3389/fpls.2018.00551 (PMC5996924; doi:10.3389/fpls.2018.00551)
Supplement: Supplementary file 1 [file Image_1.pdf]

Supplementary Material

Patterning the Asteraceae capitulum: Duplications and differential expression of the flower symmetry *CYC2*-like genes

Jie Chen[1†], Chu-Ze Shen[1†], Yan-Ping Guo[2*] and Guang-Yuan Rao[1*]

*** Correspondence:** Guang-Yuan Rao: rao@pku.edu.cn;

Yan-Ping Guo: guoyanping@bnu.edu.cn

† These authors contributed equally to this work.

Supplementary Figures

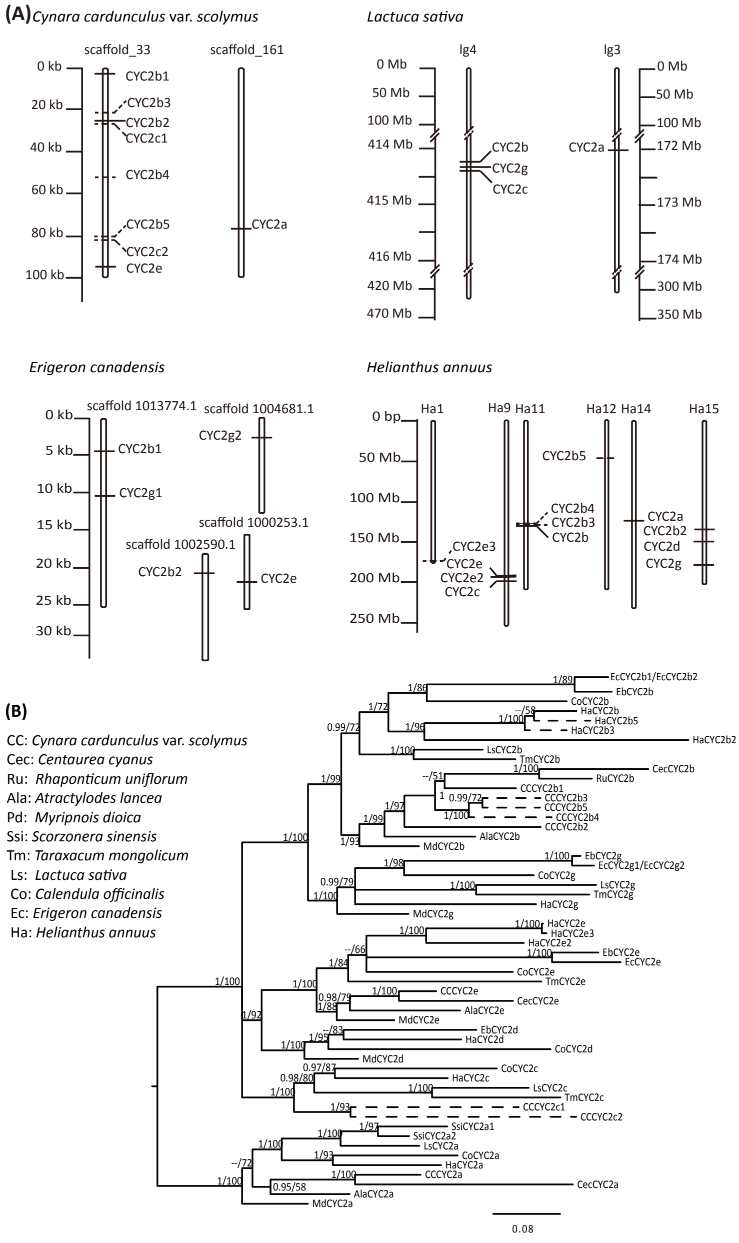

FIGURE S1. Genomic positions and relationships of *CYC2*-like genes in four Asteraceae species *Cynara cardunculus* var. *scolymus*, *Lactuca sativa*, *Erigeron canadensis*, *Helianthus annuus*, of which genomic sequencing data are available. **(A)** Mapping of *CYC2*-like genes on genomic scaffolds and chromosomes of *Cy. cardunculus* var. *scolymus*, *L. sativa*, *E. canadensis* and *H. annuus*. Dotted lines indicate pseudogenes. **(B)** Bayesian tree of *CYC* homologs found in genomes of the four species. The tree is based on nucleotide sequences aligned by MUSCLE in MEGA 7.0 (Darriba et al., 2011) under the GTR+I+G nucleotide substitution model and rooted by CYC2a clade genes. Bayesian posterior probabilities (PP > 0.95) and ML bootstrap values (> 50%) are shown on the branches. The hyphen '-' indicates PP < 0.95.

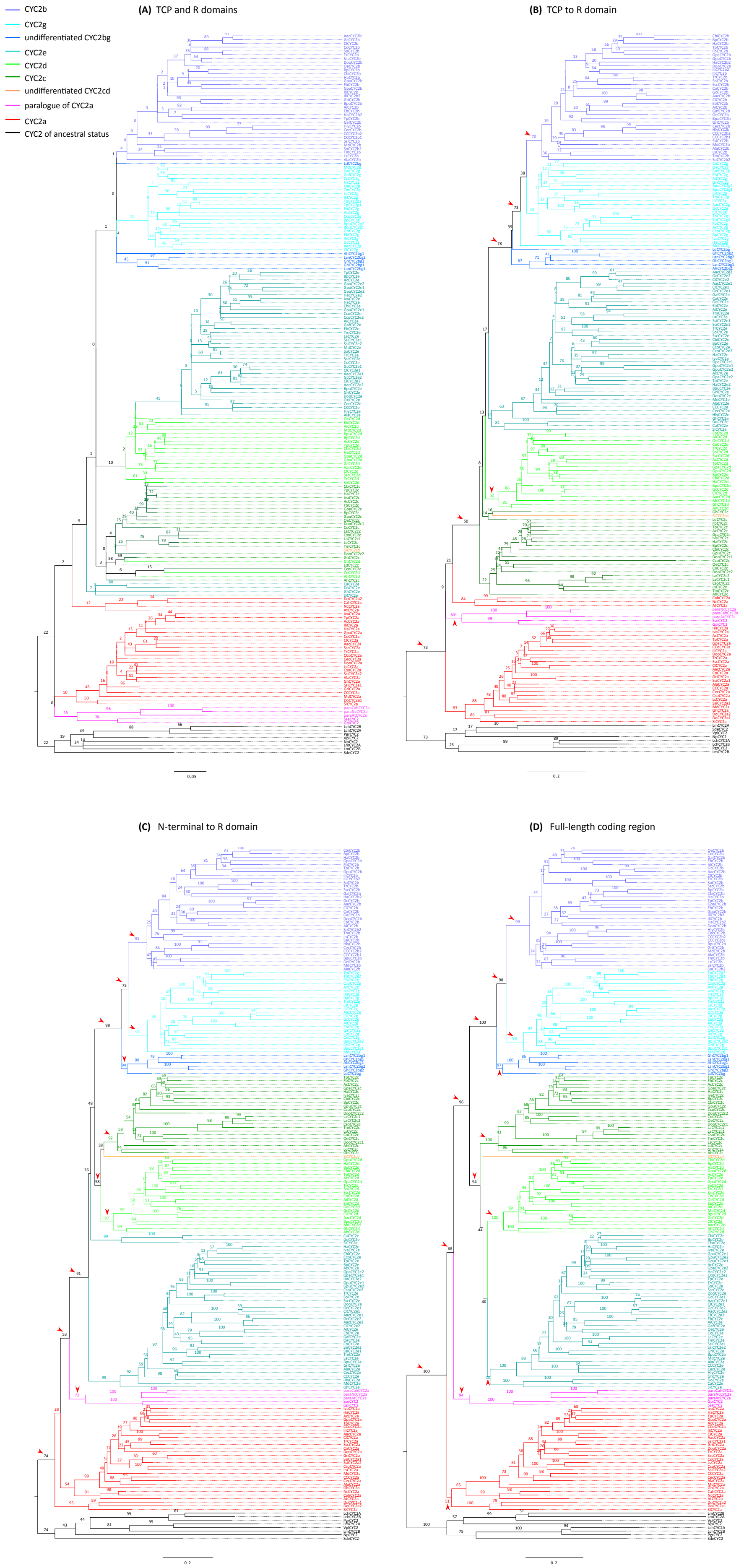
CYC2b
CYC2g
undifferentiated CYC2bg
CYC2e
CYC2d
CYC2c
undifferentiated CYC2cd
paralogue of CYC2a
CYC2a
CYC2 of ancestral status
(A) TCP and R domains
(B) TCP to R domain
(C) N-terminal to R domain
(D) Full-length coding region
0.05
0.2
0.2
0.2

FIGURE S2. Four Maximum Likelihood trees of *CYC2*-like genes in the Asterales based on different parts of 192 deduced amino acid sequences with 1000 bootstrap replicates in RAxML v8.2.9 (Stamatakis, 2014). **(A)**, **(B)** and **(C)** were reconstructed by the aligned TCP and R domain sequences, the region from TCP to R domains, and the region from the N-terminal to R domain, respectively, using JTT+I+G substitution model. **(D)** was reconstructed by the aligned full length coding region using JTT+I+G+F substitution model. Red arrowheads highlight the major branches with the bootstrap values above 50%.

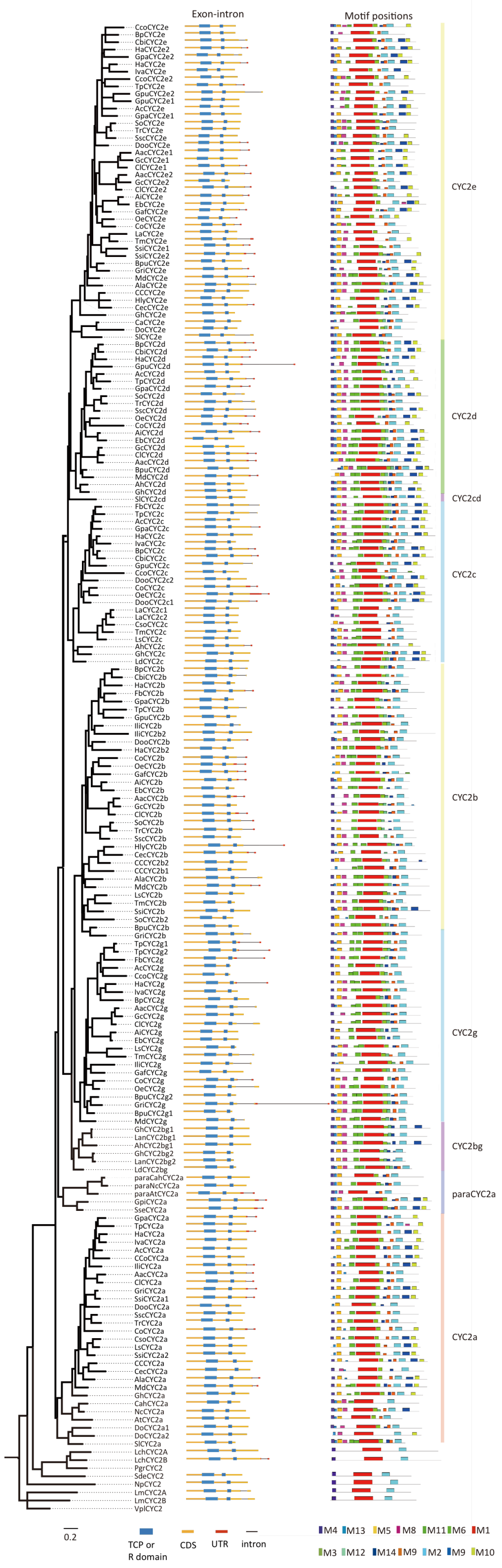

FIGURE S3. Gene tree of *CYC2*-like genes in the Asterales based on 192 deduced amino acid sequences. Each sequence is named by the initial of the species name and the gene name (for species names, see Table S1). The tree was constructed with Maximum Likelihood method (ML) and rooted by sequences of Dipsacales as the outgroup. Gene structures and putative conserved motifs are attached to each branch. Relatively conserved motifs M1~M14 (in different colors) are arranged according to their localities along the sequences. Specific motifs in CYC2a and CYC2d clades are not shown here.

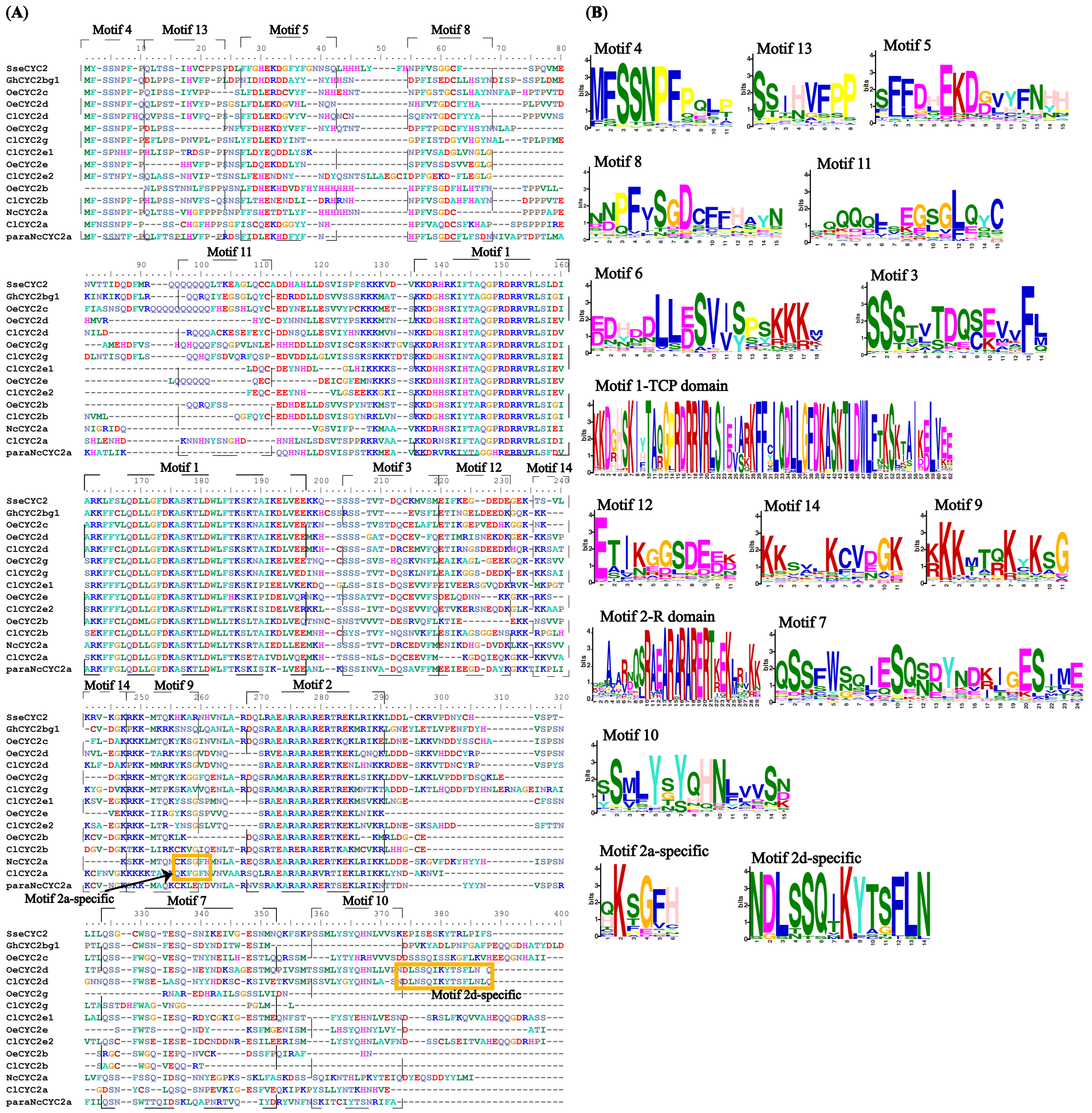

FIGURE S4. Conserved motifs of *CYC2*-like genes in the Goodeniaceae-Calyceraceae-Asteraceae (GCA) clade. **(A)** The alignment by MAFFT (Katoh and Standley, 2013) and motif distribution among fifteen representative protein sequences of *CYC2*-like genes in the GCA clade. Species names are abbreviated as follows: *Scaevola sericea* (Sse), *Nastanthus caespitosus* (Nc), *Gerbera hybrida* (Gh), *Osteospermum ecklonis* (Oe) and *Chrysanthemum lavandulifolium* (Cl)**. (B)** Fourteen conserved motifs of the majority of CYC2-like proteins in the GCA clade based on MEME test. M3, 5, and 6–14 are conserved within the GCA group. Motifs 1, 2 and 4 are conserved throughout Asterales (including a few sequences of Dipsacales) based on the comparison with all *CYC2*-like sequences from Asterales and Dipsacales. The CYC2a and CYC2d clade-specific conserved motifs are newly found during this study.

Reference:

Katoh, K., Standley, D. M. (2013). MAFFT Multiple Sequence Alignment Software Version 7: improvements in Performance and Usability. Mol. Biol. Evol. 30, 772–780. doi: 10.1093/molbev/mst010

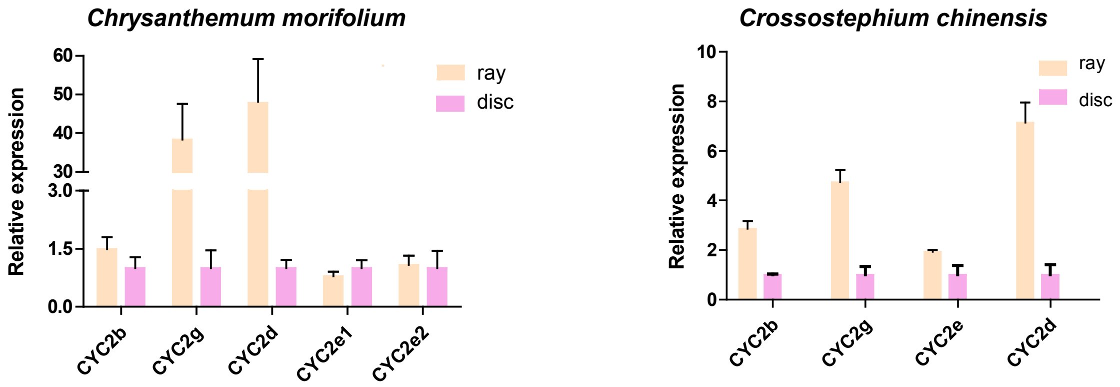

FIGURE S5. Relative expressions of the Asteraceae *CYC2*-like genes in *Chrysanthemum morifolium* and *Crossostephium chinensis*. Expression levels of *ACTIN7* were used for normalization; relative expression levels of each *CYC2* copy in all samples were normalized against its expression in the disc florets via the $2^{-\Delta\Delta C_T}$ method (Livak and Schmittgen, 2001). Error bar is ± s.e. value of three biological samples.

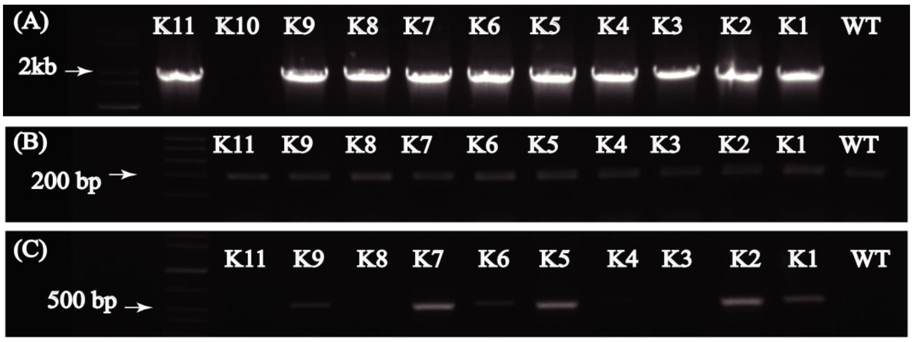

FIGURE S6. Verification of 35s::*ClCYC2d* in the transgenic lines of *C. lavandulifolium* by amplifications using primer pairs **(A)** PLACF/LACR (35 PCR cycles), **(B)** Actin7F/R (22 PCR cycles), and **(C)** 35spA-r/ClCYC2d-sf (32 PCR cycles).

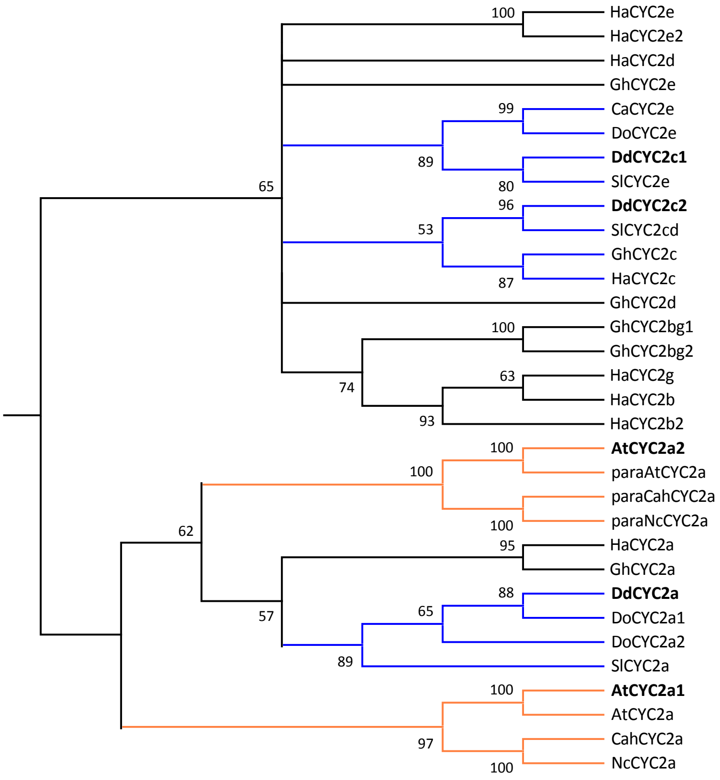

FIGURE S7. Relationships of *CYC2*-like homologs in the family Calyceraceae (orange branches) and the subfamily Barnadesioideae of Asteraceae (blue branches) isolated during this study and by Chapman et al. (2012; gene names are shown in bold letters). The tree was constructed by ML method under the GTR+I+G nucleotide substitution model, based on nucleotides sequences between the TCP and R domains aligned by MUSCLE in MEGA 7.0 (Darriba et al., 2011). Species names are abbreviated as follows: *Acicarpha tribuloides* (At), *Nastanthus caespitosus* (Nc), *Calycera herbacea* (Cah) from Calyceraceae; *Chuquiraga aurea* (Ca), *Doniophyton* sp. (Do), *Dasyphyllum diacanthoides* (Dd) and *Schlechtendalia luzulaefolia* (Sl) from Barnadesioideae; and *Gerbera hybrida* (Gh) and *Helianthus annuus* (Ha). Accession numbers of sequences from NCBI: JF299240–1; JF299246–8; U429303–EU429305, JN190059, JN190061, JN190063; EU088368–72.
