# Supplementary material for: Patterning the Asteraceae Capitulum: Duplications and Differential Expression of the Flower Symmetry CYC2-Like Genes
Source: Front Plant Sci. 2018 Apr 25;9:551. doi: 10.3389/fpls.2018.00551 (PMC5996924; doi:10.3389/fpls.2018.00551)
Supplement: Supplementary file 2 [file Image_2.PDF]

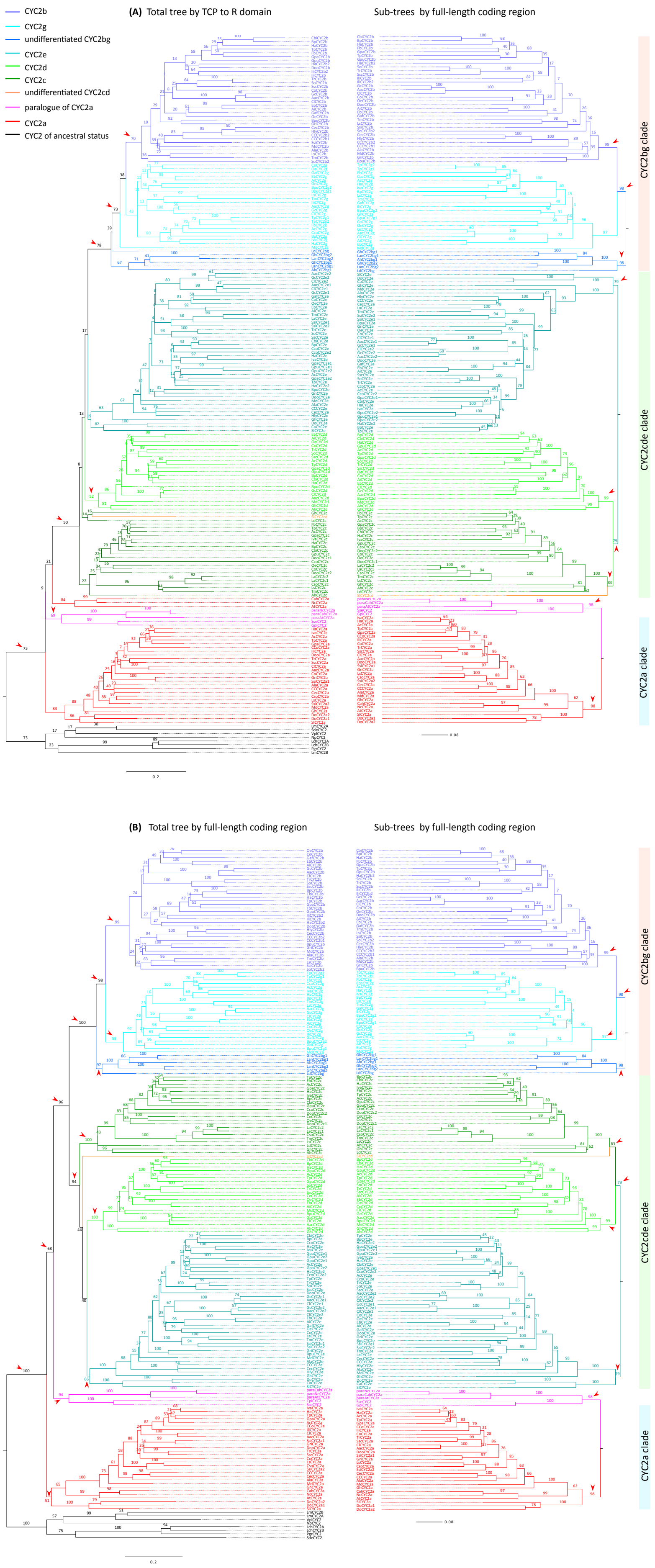

Additional Figure

"Total" trees constructed on the complete dataset of the Asterales *CYC2* genes (left side) are compared against three sub-trees on different groups of the *CYC2* genes, i.e., the CYC2a, CYC2bg and CYC2cde groups (right side). All the trees were constructed with 1000 bootstrap replicates in RAxML v8.2.9 (Stamatakis, 2014). The "total" trees were reconstructed using JTT+I+G+F (for **A**) and JTT+I+G (for **B**) substitution models. Three sub-trees were reconstructed with the full-length alignments using JTT+I+G substitution model. Red arrowheads highlight the major branches with bootstrap values above 50%.
