# Supplementary material for: Patterning the Asteraceae Capitulum: Duplications and Differential Expression of the Flower Symmetry CYC2-Like Genes
Source: Front Plant Sci. 2018 Apr 25;9:551. doi: 10.3389/fpls.2018.00551 (PMC5996924; doi:10.3389/fpls.2018.00551)
Supplement: Supplementary file 4 [file Table_2.docx]

Supplementary Material

Patterning the Asteraceae capitulum: Duplications and differential expression of the flower symmetry CYC2-like genes

**Jie Chen^1†^, Chu-Ze Shen^1†^, Yan-Ping Guo^2^**^*^ **and Guang-Yuan Rao^1^**^*^

*** Correspondence:** Guang-Yuan Rao: [rao@pku.edu.cn](mailto:rao@pku.edu.cn);

Yan-Ping Guo: [guoyanping@bnu.edu.cn](mailto:guoyanping@bnu.edu.cn)

**†** These authors contributed equally to this work.

**Supplementary Tables**

**TABLE S1.** Species samples and categories of the *CYC2*-like genes analyzed by this study (This is provided separately in a Microsoft Excel file)

**TABLE S2.** Primers for amplification of the *CYC2*-like genes during this study.

| Name | Sequences（5’－3’） | Application |
| --- | --- | --- |
| oligo(dT)_18_ | GCTGTCAACGATACGCTACGTAACGGCATG | Reverse transcription primer for first strand cDNA |
|  | ACAGTGTTTTTTTTTTTTTTTTTT |  |
| CYC2-3rF1 | AKTTGCTAGGKTTTGAYAAAGCAAG | CYC2-specific forward primer for 3’RACE |
| CYC2-3rF2 | GCAAGCAAAACCCTTGATTGGYT | CYC2-specific froward primer for 3’RACE |
| GSP1 | GCTGTCAACGATACGCTACGTAACG | Universal reverse primer for 3’RACE |
| GSP2 | CGCTACGTAACGGCATGACAGTG | Universal reverse primer for 3’RACE |
| CYC2-ATG | ATGTTTTCCTCAAACCCTTT | Conserved primer for CYC2a clade genes |
| CYC2a-ATG | ATGTTTAATTCTTCAAACCCT | Conserved primer for CYC2 clade genes except CYC2a |
| AAP | GGCCACGCGTCGACTAGTACGGGIIGGGIIGGGIIG | Universal forward primer for 5'RACE |
| AUAP | GGCCACGCGTCGACTAGTAC | Universal forward primer for 5'RACE |
| AP1 | GTAATACGACTCACTATAGGGC | Universal primer for Genome walking |
| AP2 | ACTATAGGGCACGCGTGGT | Universal primer for Genome walking |
| GWadaptorF | GTAATACGACTCACTATAGGGCACGCGTGGTCGACGATTCCTGGG | Adaptor for genome walking |
| GWadaptorR | PO4-CCCAGGAAT-3ddC | Adaptor for genome walking |
| CTf | AARGAYAGGCACAGCAA | Chapman et al., 2008 |
| CTr | TCCTTRGTYCKYTCCCT | Chapman et al., 2008 |
| CYC2a-r1 | TTGAKTCAACAWAGTTGTGSTGG | Conserved primer for CYC2a clade genes |
| CYC2a-r2 | CATWGAGTATGGYTTTGGAAGCTT |  |
| CYC2d-r | TGYAAATTTAGGAAACTTGTGTACT | Conserved primer for CYC2d clade genes |
| pobCYC2d-r1 | CCTCTTGTAAATCTAAACACTGC | Specific primer for CYC2d for *Gnaphalium affine* |
| pobCYC2d-r2 | GGTAAACAATAGATTTGTGTGTCAG |  |

Note: Amplifications of *CYC2a* and *CYC2d* genes in the genomic DNA of *Gnaphalium affine* and five Cichorieae species used randomly combined forward and reverse primers. The forward primers CYC2-ATG, CYC2-3rF1, CYC2-3rF2 and CTf and the reverse primer CTr are fitting for all *CYC2*-like genes in Asteraceae. The forward primers CYC2a-ATG and the reverse primers CYC2a-r1/2 are specific to *CYC2a* genes. The reverse primer CYC2d-r is specific to *CYC2d* genes). The reverse primers pobCYC2d-r1 and 2 from *Pseudognaphalium obtusifolium* are specific to *CYC2d* of *Gnaphalium affine*.

**TABLE S3.** *CYC2*-like homologs from the whole-genome sequencing database.

| Gene | Scaffold No. | Location | Integrity | Normality |
| --- | --- | --- | --- | --- |
| *Cynara cardunculus* var. *scolymus*, *genotype ‘2C’* | | | | |
| CCCYC2a | 161 | 76981–77973 | Complete | normal translation |
| CCCYC2b1 | 33 | 2938–3888 | Complete | normal translation |
| CCCYC2b2 | 33 | 24173–25141 | Complete | normal translation |
| CCCYC2b3 | 33 | 21597–22578 | Complete | pseudogene |
| CCCYC2b4 | 33 | 52297–53136 | Complete | pseudogene |
| CCCYC2b5 | 33 | 80283–81155 | Complete | pseudogene |
| CCCYC2c1 | 33 | 26672–27878 | Complete | pseudogene |
| CCCYC2c2 | 33 | 82303–83103 | partial | pseudogene |
| CCCYC2e | 33 | 86400–95376 | Complete | normal translation |
| *Lactuca sativa* | | | | |
| LsCYC2a | lg3 | 172213484–172214386 | complete | normal translation |
| LsCYC2b | lg4 | 414236094–414236996 | complete | normal translation |
| LsCYC2c | lg4 | 414380769–414381620 | complete | normal translation |
| LsCYC2g | lg4 | 414328113–414328929 | complete | normal translation |
| *Conyza canadensis* (*Erigeron canadensis*) | | | | |
| EcCYC2b1 | 1013774.1 | 4430–5245 | complete | normal translation |
| EcCYC2b2 | 1002590.1 | 24533–3348 | complete | normal translation |
| EcCYC2e | 1000253.1 | 6143–7063 | complete | normal translation |
| EcCYC2g1 | 1013774.1 | 11193–12422 | complete | normal translation |
| EcCYC2g2 | 1004681.1 | 2461–3690 | complete | normal translation |
| *Helianthus annuus* | | | | |
| HaCYC2a | Ha14 | 12377320–12378231 | complete | normal translation |
| HaCYC2b | Ha11 | 129782348–129781545 | complete | normal translation |
| HaCYC2b3 | Ha11 | 129779630–12779291 | partial | pseudogene |
| HaCYC2b4 | Ha11 | 126908472–126907769 | partial | pseudogene |
| HaCYC2b5 | Ha12 | 46345646–46346387 | partial | pseudogene |
| HaCYC2b2 | Ha15 | 133966601–133967341 | complete | normal translation |
| HaCYC2c | Ha9 | 197693449–197694522 | complete | normal translation |
| HaCYC2d | Ha15 | 148310108–148309191 | complete | normal translation |
| HaCYC2e | Ha9 | 199043136–199044009 | complete | normal translation |
| HaCYC2e2 | Ha9 | 199073862–199074788 | complete | pseudogene |
| HaCYC2e3 | Ha1 | 175643771–175643377 | partial | normal translation |
| HaCYC2g | Ha15 | 178543876–178544649; | complete | normal translation |
|  |  | 178545086–178545152 |  |  |

**TABLE S4.** Primers for RT-qPCR experiments.

| Species | Gene | Forward primer 5’-3’ | Reverse primer 5’-3’ |
| --- | --- | --- | --- |
| *Taraxacum* | Actin7 | GTCCCACACTGTTCCAATTTAC | CGTAGTCGAGAGCCACATAAG |
| *mongolicum* | CYC2b | CATTAATGGTGGAATAATCGAGGA | GTCTTCAATTCATCATTAACACGCA |
|  | CYC2c | CTCCTTCTTTGACCACGACATA | CATCACCGTACTGCAACAAAC |
|  | CYC2e | CCTCCTCCGAATTCGTTTCTT | CTTGTTCTAACCCTAATCCTTCGT |
|  | CYC2g | CCAATAATGTCTTCTTTCCACCCA | GACTGCAACCCTAGTTCTTCTTC |
|  |  |  |  |
| *Chrysanthemum* | Actin7 | TCCGGCTATGTATGTTGCTATTC | AATCTTCATCAAGGGATCGGTAAG |
| *morifolium* | CYC2b | CCATTGCTCATATTCCACAGTTTAT | CTCTGTCCTTGACTGAYCTCTTG |
|  | CYC2d | CCCAGTTCTTTCTTTGACCTTG | ACTGACTCTAGAAGTTGACTGTTAT |
|  | CYC2e1 | CAGTTCTGGATCTCCCATGAATC | CCCAATCTTCCCACAATAATCTCT |
|  | CYC2e2 | GCAAGAGCTAGAGAAAGGACTAA | GCAAGGTTGTGCTGATAACTATAC |
|  | CYC2g | CAGCAATCAAGTATGGTGATGTC | CTCCTGCATTTCTTTCCAAGTT |
|  |  |  |  |
| *Crossostephium* | Actin7 | ATGGCCGACACCGAAGATATTC | GATACCTCTCTTTGATTGTGCTTCA |
| *chinensis* | CYC2b | CTGGATCAGGCGGAGAAAAT | CAACCATGATGAAGCCTCTTAAC |
|  | CYC2d | GCACCAAAGGAAGAAGTCAGTA | GGCTTACAGGGCGATAACAA |
|  | CYC2e1 | AGAACGATCGGATGTACAAGAAA | GAACATTCTCCGTCGAGCTT |
|  | CYC2e2 | TCATCTTCCACTGTGACTGTTC | GATAGCCCTAGACTGGTCTCTT |
|  |  |  |  |
| *Chrysanthemum* | Actin7 | TCCGGCTATGTATGTTGCTATTC | AATCTTCATCAAGGGATCGGTAAG |
| *lavandulifolium* | CYC2a | GCAGCTAGAAGCCAGTTAAGA | GTTCTGCAAACGATTCCCTAATTT |
|  | CYC2b | GACTACTCGATGGTGTCGATG | GTACTTTGCTGTTCCACTTGAC |
|  | CYC2d | GAGGAAGAGGTCAGCAACTAAG | GGCTTACAGGGCGATAACA |
|  | CYC2e1 | TCCCAGAAATCGTTGAAGAAC | AACATTCTCCGTTGAGCTTT |
|  | CYC2e2 | GGCTCGATAATGAGTCCAAGAG | TCAGACAAGCAGCTCGAATC |
|  | CYC2g | CAGCAATCAAGTATGGTGATGTC | CTCCTGCATTTCTTTCCAAGTT |
|  | 2dUTR | TTGGAGTGAACTTGCATCTCA | CAGCCAATAATTCTCTTGTGGAC |
|  |  |  |  |
| *Calendula* | Actin7 | CCTTCAATGTCCCTGCTATGT | GTGACACACCATCACCAGAAT |
| *officinalis* | CYC2b | CAATTTATCTCCGGCGACTTTC | GAATACTGCAACCCTGATCCT |
|  | CYC2c | TCATGCTTTCCCTCCATCTTC | GAAACTGCTGTTGTTGCCTAAG |
|  | CYC2d | GTCCTCCCTCCTACCGATTAT | CCGTTGACAAAGTGGTTGTTG |
|  | CYC2e | AGCAACAACCCATGTGTCTC | TCACACTGTTGCAACCCTAATC |
|  | CYC2g | GGAAGGAAATGCAGGAGAAGA | TACTCCAGATCCACCAACTACT |

**TABLE S4.** Continued.

| Species | Gene | Forward primer 5’-3’ | Reverse primer 5’-3’ |
| --- | --- | --- | --- |
| *Osteospermum* | Actin7 | CTCACTGAGGCACCTCTAAATC | AGGTAAAGCATAACCCTCGTAAA |
| *ecklonis* | CYC2b | CCACCAATAATCTCTTCTCTCCTC | TGTGTTGTAAGGAGACACTACAG |
|  | CYC2c | CAGATTCCCTCTTCCATCTATGTC | TTGTTGTTGTTGCTGCCTAAC |
|  | CYC2d | CCTCCTTCCGGTTCTTTGTTTG | GACCATCTTTCTTCAAGTTTGTCAT |
|  | CYC2e | CAGATTCCCTCTTCCATCTATGTC | TTGTTGTTGTTGCTGCCTAAC |
|  | CYC2g | CAACACCGACCCGTTTAC | CGATTTCGAGCAAGAAATTACC |
|  |  |  |  |
| *Tagetes* | Actin7 | TGACAATGGAACTGGGATGG | CGATACCGTGCTCAATAGGATAC |
| *patula* | CYC2b | AGAGATCAGTTAAGGGCAGAGG | GCTGCTCATGAAATGCTGATCTA |
|  | CYC2c | CCAGTTTGTGTCTGACTCTAATTTC | GATGGGTATACTACTGACTCCAAA |
|  | CYC2d | TCCTCCTTCTACTACCTCTTTCT | TGGCTGCCTAACCATGATATT |
|  | CYC2e | GCTTGATGATGAGTCCAACACTA | GTTGCACAATCACCTTGCTG |
|  | CYC2g | CAGTTTGCTCAAGGACCATTT | GACAATCTCACTCTCCGATCTC |
|  |  |  |  |
| *Bidens* | Actin7 | GTATTGTCAGCAACTGGGATGA | GAGGTGCTTCGGTTAGGAGAAC |
| *pilosa* | CYC2b | GCCAAGACTTAAATGTGTAGAT GGG | CTCAAGATCCCTGAAAGCTTGAATC |
|  | CYC2c | CTCGACAACGAGTCGAAGAAA | TGGGAGATCCACTCATCTGATA |
|  | CYC2d | GAAAGGACGGGTGAGTCAATTA | CTTGGTGGAGATCACAATCTTCT |
|  | CYC2e | CAAGTTCACAGCTTTGCCTAAA | ACTCACAATACCAATCCAGCTA |
|  | CYC2g | GCTGGAGCTCAAGAGAATCAT | CACTCGATATCCGCCTTTAACT |
|  |  |  |  |
| *Helianthus* | Actin7 | GTATTGTCAGCAACTGGGATGA | ATGGTGCCTCAGTGAGAAGAAC |
| *annuus* | CYC2b | GGCTCTTCACCAAGTCTCTAACAG | GGCTCTTTGAGTCCCTTTCTTCC |
|  | CYC2c | CCCTCAAACACAACACTCCAATC | TGACTCTGCTCCTCGTGAATTAC |
|  | CYC2d | TACCACCACCACCACAACAAC | GACATCCTGACCCTCCT ATCTC |
|  | CYC2e | AACCGACGAACAAGCGAAAG | TGAAGAACAGCAGTCAACAGG |
|  | CYC2g | ACTAGTTCCTGATGAGTTTGACTG | TGGTAGACTTACATCCTTACAATATCAC |

Note: RT-qPCR primers for *CYC2b*~*CYC2e* and *Actin7* genes in *Helianthus annuus* are from Tähtiharju et al. (2012).

**TABLE S5.** Primers used in the analysis of transgenic lines.

| Name | Sequences 5'-3' |
| --- | --- |
| Constructing binary plasmids | |
| K_CaMV_35s_f | CGGGGTACCAGTCTCAGAAGACCAAAGGGCT |
| 35S_Cl2d_r | TTGAGGAAAACATGTAGAGAGAGACTGGTGATTTCAG |
| 35S_Cl2d_f | CTCTCTCTACATGTTTTCCTCAAACCCATTTCATC |
| Cl2d_pA_r | CGAGCTTGTCGACTACTGTAAATTTAGGAAACTTGTGTACTTG |
| Cl2d_pA_f | AATTTACAGTAGTCGACAAGCTCGAGTTTCTCCAT |
| B_ployA_r | CGCGGATCCGATCTGGATTTTAGTACTGGATTTTGGT |
|  |  |
| Checking target recombinant in the genome | |
| PLACF | GCGCAACGCAATTAATGTGAGTT |
| LACR | TTAAGTTGGGTAACGCCAGGGTT |
|  |  |
| Checking target recombinant in transcripts | |
| 35spA-r | TCCCTTATCTGGGAACTACTCAC |
| ClCYC2d-sf | TCATCTTCTAGTGCTACGGATCG |

**TABLE S6.** Lineage-specific replicates of some CYC2 clade genes in Asteraceae.

| Species | Gene of replicates | Identity/% | Similarity/% | NO. of introns |
| --- | --- | --- | --- | --- |
| *Doniophyton* | DoCYC2a1/2a2 | 53.15 | 54.43 | -/- |
| *Cynara cardunculus* | CcCYC2b1/2b2 | 57.46 | 56.41 | 0/0 |
| var. *scolymu* |  |  |  |  |
| *Scorzonera sinensis* | SsiCYC2e2/2e2 | 67.43 | 68.88 | 1/1 |
| *Berkheya purpurea* | BpuCYC2g1/2g2 | 66.26 | 68.65 | -/- |
| *Doronicum orientale* | DooCYC2c1/2c2 | 72.53 | 72.02 | 0/1 |
| *Sinosenecio oldhamianus* | SoCYC2b/2b2 | 54.9 | 53.22 | 1/0 |
| *Chrysanthemum lavandulifolium* | IliCYC2b/2b2 | 61.53 | 64.47 | 1/1 |
| *Achillea acuminata* | AacCYC2e1/2e2 | 57.73 | 59.74 | 1/1 |
| *Inula lineariifolia* | IliCYC2b/2b2 | 69.2 | 49.14 | 0/0 |
| *Galinsoga parviflora* | GpaCYC2e1/2e2 | 64.02 | 63.1 | 0/1 |
| *Helianthus annuus* | HaCYC2e/2e2 | 74.58 | 76.45 | 1/1 |
| *Helianthus annuus* | HaCYC2b/2b2 | 53.03 | 51.37 | 0/0 |
| *Tagetes patula* | TpCYC2g1/2g2 | 91.12 | 92.6 | 1/1 |
| *Conoclinium coelestinum* | CcoCYC2e1/2e2 | 61.78 | 59.22 | -/- |

Notes: The hyphen “-” stands for no information. The identity and similarity of the potentially paralogous pairs were calculated by an online tool “Sequence Identity And Similarity” on the website <http://imed.med.ucm.es/Tools/sias.html>.
